# Development of a therapeutic anti-HtrA1 antibody and the identification of DKK3 as a pharmacodynamic biomarker in geographic atrophy

**DOI:** 10.1073/pnas.1917608117

**Published:** 2020-04-28

**Authors:** Irene Tom, Victoria C. Pham, Kenneth J. Katschke, Wei Li, Wei-Ching Liang, Johnny Gutierrez, Andrew Ah Young, Isabel Figueroa, Shadi Toghi Eshghi, ChingWei V. Lee, Jitendra Kanodia, Scott J. Snipas, Guy S. Salvesen, Phillip Lai, Lee Honigberg, Menno van Lookeren Campagne, Daniel Kirchhofer, Amos Baruch, Jennie R. Lill

**Affiliations:** ^a^OMNI Biomarker Development, Genentech, Inc., South San Francisco, CA 94080;; ^b^Department of Microchemistry, Proteomics & Lipidomics, Genentech, Inc., South San Francisco, CA 94080;; ^c^Department of Immunology, Genentech, Inc., South San Francisco, CA 94080;; ^d^Department of Early Discovery Biochemistry, Genentech, Inc., South San Francisco, CA 94080;; ^e^Department of Antibody Discovery, Genentech, Inc., South San Francisco, CA 94080;; ^f^Drug Metabolism, Pharmacokinetics, and Bioanalysis, AbbVie, South San Francisco, CA 94090;; ^g^Biology Core Support, Gilead Sciences, Foster City, CA 94404;; ^h^Clinical and Translational Pharmacology, Theravance Biopharma, Inc., South San Francisco, CA 94080;; ^i^National Cancer Institute-Designated Cancer Center, Sanford Burnham Prebys Medical Discovery Institute, La Jolla, CA 92037;; ^j^Early Clinical Development OMNI Department, Genentech, Inc., South San Francisco, CA 94080;; ^k^Inflammation & Oncology Research, Amgen, South San Francisco, CA 94080;; ^l^Biomarker Development, Calico Life Sciences, LLC, South San Francisco, CA 94080

**Keywords:** age-related macular degeneration (AMD), proteomics, biomarker

## Abstract

Genetic polymorphisms in the region of the trimeric serine hydrolase high-temperature requirement 1 (*HTRA1*) are associated with increased risk of age-related macular degeneration (AMD) and disease progression, but the precise biological function of HtrA1 in the eye and its contribution to disease etiologies remain undefined. In this study, we have developed an HtrA1-blocking Fab fragment to test the therapeutic hypothesis that HtrA1 protease activity is involved in the progression of AMD. Next, we generated an activity-based small-molecule probe (ABP) to track target engagement in vivo. In addition, we used N-terminomic proteomic profiling in preclinical models to elucidate the in vivo repertoire of HtrA1-specific substrates, and identified substrates that can serve as robust pharmacodynamic biomarkers of HtrA1 activity. One of these HtrA1 substrates, Dickkopf-related protein 3 (DKK3), was successfully used as a biomarker to demonstrate the inhibition of HtrA1 activity in patients with AMD who were treated with the HtrA1-blocking Fab fragment. This pharmacodynamic biomarker provides important information on HtrA1 activity and pharmacological inhibition within the ocular compartment.

## Significance

Genome-wide association studies have identified genetic variation at the *ARMS2/HTRA1* locus as a risk factor for the development and progression of age-related macular degeneration (AMD). We have developed a potent anti-HtrA1 Fab inhibitor of HtrA1 proteolytic activity in the retina as a potential therapeutic for treating AMD. A set of proteomic analytical tools was established to characterize HtrA1 activity and discover in vivo HtrA1 substrates. These efforts led to the identification of an eye-specific and clinically applicable pharmacodynamic biomarker of anti-HtrA1 Fab activity. Analysis of HtrA1-mediated cleavage of Dickkopf-related protein 3 in the aqueous humor of patients with geographic atrophy provided evidence of anti-HtrA1 Fab activity and information on duration of activity in a phase 1 study.

Age-related macular degeneration (AMD) is a common ocular disease that affects the macular region of the retina, causing progressive loss of central vision. It is the leading cause of visual impairment in the developed world (1, 2). The early stage of AMD is characterized by the accumulation of drusen droplets containing lipids and proteins at the boundary between the retinal pigment epithelium (RPE) and Bruch’s membrane. Intermediate AMD can further progress into two distinct forms of symptomatic advanced disease: neovascular AMD (wet AMD), characterized by the invasion of blood vessels into the neural retina, and geographic atrophy (GA), characterized by the loss of photoreceptors, RPE, and the choriocapillaries (3). The risk of developing AMD is influenced by age, environmental factors such as smoking and diet, and genetics. Genome-wide association studies have identified several risk loci that may contribute to the initiation and progression of AMD (4). One such major risk locus at 10q26 encompasses three genes: 1) pleckstrin homology domain-containing A1 (*PLEKHA1*), 2) age-related maculopathy susceptibility 2 (*ARMS2*), and 3) high-temperature requirement 1 (*HTRA1*) with high linkage disequilibrium. Hence, the individual contribution of the encoded proteins to disease etiology remains unclear. Genetic variation at this locus, also termed the *ARMS2/HTRA1* locus, increases the risk for both neovascular AMD and GA (4). ARMS2 messenger RNA is only expressed in humans and chimpanzees, and its biological relevance to AMD is not well-understood (5). HtrA1 protein is expressed in the RPE and in horizontal cells in the human retina (6). It contains a protease domain with a trypsin-like fold with a catalytic triad composed of His220, Asp250, and the active-site nucleophile Ser328. In addition to the catalytic domain, HtrA1 contains several functional domains including an N-terminal insulin-like growth factor-binding protein/Kazal domain and a C-terminal PDZ domain (post synaptic density protein [PSD95], Drosophila disc large tumor suppressor [Dlg1], and zonula occludens-1 protein [ZO-1]) (7). Loss-of-function mutations in the HtrA1 protease domain lead to cerebral autosomal recessive arteriopathy with subcortical infarcts and leukoencephalopathy (8). Differential HtrA1 expression or mutation is implicated in tumorigenesis as well as autoimmunity (9, 10). HtrA1 can cleave a plethora of substrates such as transforming growth factor beta, fibronectin, amyloid precursor protein, and other extracellular matrix proteins (10–13). The relevance of these putative substrates to HtrA1 biology in nonengineered in vivo settings has yet to be established.

Given that HtrA1 activity appears to be potentially linked to AMD pathology, we have designed an inhibitory anti-HtrA1 Fab to investigate the consequence of HtrA1 inhibition in the context of this ocular disease. To assess the inhibitory effects of this antibody in preclinical and clinical applications, we have developed an HtrA1-directed activity-based profiling probe and performed an N-terminomic proteomic approach to identify HtrA1 substrates as potential biomarkers. A two-pronged in vivo proteomic approach yielded three ocular substrates that were consistently identified in independent cross-species studies. One of these substrates, Dickkopf-related protein 3, was shown to be a robust pharmacodynamic biomarker for anti-HtrA1 activity in preclinical animal models and, most notably, a clinically applicable biomarker for anti-HtrA1 in a phase 1 study in GA patients.

## Results

### Generation and Characterization of Anti-HtrA1 Antibodies.

Anti-HtrA1 antibodies were obtained by using recombinant HtrA1 protease domain (HtrA1-PD) for immunization of HtrA1-knockout mice, which were generated by traditional homologous recombination methods. Using standard hybridoma methods, we identified 74 clones that bound to human HtrA and one of them, clone 15H6, showed strong inhibition of human HtrA1 enzymatic activity. The mouse Fab15H6 was subsequently humanized by grafting the hypervariable regions into a human consensus framework while retaining key murine residues at the Vernier zone. The obtained Fab15H6.v2 was modified by changing two problematic residues, N94A (cleavage) and D55 (isomerization), and subsequently affinity-matured by using Fab phage display combined with deep-sequencing analysis. As compared with Fab15H6.v2, the obtained Fab15H6.v4 had a total of four changes: N94A (complementarity-determining region [CDR] L3), D55E (CDR-H2), N31E (CDR-L1), and T28K (CDR-H1). To eliminate the immunogenicity potential of the exposed upper hinge region of the heavy chain, we deleted the C-terminal residues K222 to T225 to produce the final Fab15H6.v4.D221 ending with residue D221.

**Fig. 1.**
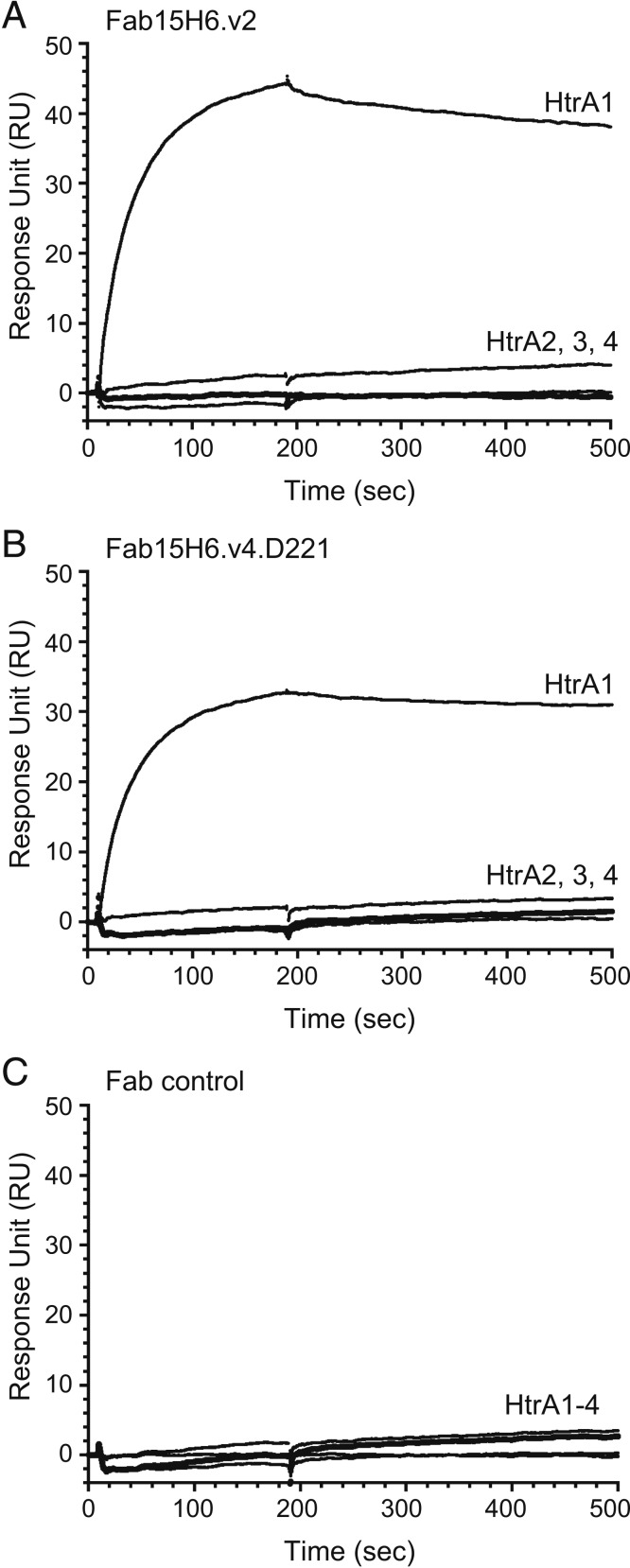
Binding specificity of Fab15H6.v2 and Fab15H6.v4.D221 as measured by surface plasmon resonance. The His-tagged trimers of HtrA1-PD/PDZ, HtrA2-PD/PDZ, HtrA3-PD/PDZ, and HtrA4-PD/PDZ (labeled HtrA1, 2, 3, and 4, respectively) were captured by anti-His tag sensor chip CM5 on a BIAcore T200 instrument, followed by injecting (100 nM) either Fab15H6.v2 (*A*) or Fab15H6.v4.D221 (*B*), or a control Fab (anti-PCSK9 Fab33) (*C*), to measure the binding responses. The Fabs specifically bound to HtrA1-PD/PDZ and did not bind to any other HtrA family member at a Fab concentration of 100 nM, which was 170-fold above the determined *K*_D_ value for HtrA1-PD.

The specificity and affinity of Fab15H6.v2 and Fab15H6.v4.D221 were determined, since these antibodies were subsequently used for studies in rabbit and cynomolgus monkey, respectively. The *Escherichia coli*-expressed and purified human HtrA1 to 4 constructs comprising the protease domain (PD) and PDZ domains (HtrA1- to 4-PD/PDZ) were purified as homotrimers by size-exclusion chromatography (*SI Appendix*, Fig. S1*A*). Surface plasmon resonance experiments demonstrated that the antibodies were highly specific, since they only bound to HtrA1-PD/PDZ but not to the other HtrA family members (Fig. 1). Furthermore, both antibodies had strong binding affinities for HtrA1-PD/PDZ, the determined *K*_D_ values being 0.559 and 0.588 nM for Fab15H6.v2 and Fab15H6.v4.D221, respectively (*SI Appendix*, Fig. S1*B*).

### Development and Characterization of an Activity-Based Small-Molecule Probe for HtrA1.

**Fig. 2.**
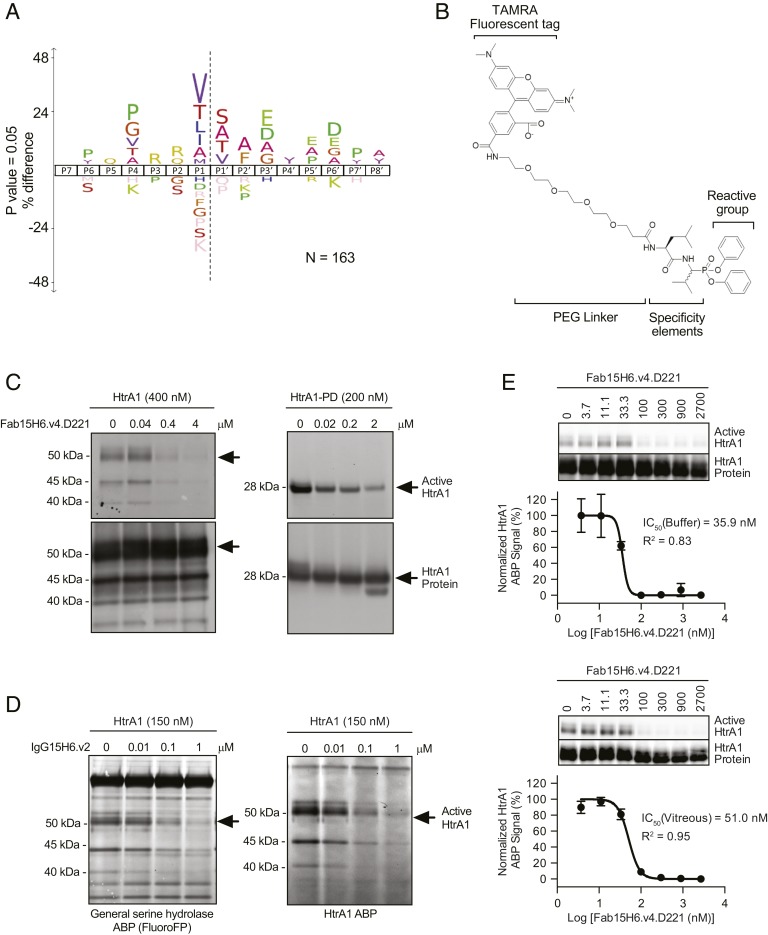
Design and characterization of a specific ABP for HtrA1. (*A*) The consensus sequence specificity of HtrA1 proteolytic cleavage of rat vitreous substrates in vitro was determined by N-terminomics and revealed a preference for hydrophobic residues at the P1 position in a variety of substrates present in the vitreous. The number of peptides used to generate the sequence logo was 163 and the iceLogo was generated using *Rattus* species as a reference set with a *P* value of 0.05. (*B*) Probe structure. (*C*) Preincubation of either HtrA1 or HtrA1-PD with a monoclonal antibody that inhibits proteolytic activity of HtrA1 (Fab15H6.v4.D221) blocked activity-based probe labeling in a concentration-dependent manner. Probe labeling was assessed by SDS/PAGE and visualized by fluorescent gel imaging (*Top*). Total protein was detected by immunoblotting with biotinylated anti-HtrA1:7816 19G10 followed by horseradish peroxidase-conjugated streptavidin (*Bottom*). Arrows indicate intact HtrA1 or HtrA1-PD on fluorescent gel images or immunoblots, respectively. Lower–molecular-mass bands are likely the result of HtrA1 autolytic activity and were determined to be cleaved HtrA1 migrating at different positions by N-terminal sequencing. (*D*) To evaluate ABP labeling in complex proteomes, HtrA1 was added to rabbit vitreous humor followed by labeling with a general serine hydrolase ABP (fluoroFP) or the HtrA1 ABP. While fluoroFP labeled many bands in vitreous humor, corresponding to the expected array of vitreal endogenous serine hydrolases, the HtrA1 ABP selectively labeled HtrA1 and labeling could be inhibited by preincubation with anti-HtrA1 IgG15H6.v2. Arrows indicate intact HtrA1 bands that were labeled by fluoroFP or HtrA1 ABP. Lower–molecular-mass bands were determined to be cleaved HtrA1 by immunoprecipitation with anti-HtrA1 antibody. (*E*) Inhibition of HtrA1 ABP labeling by anti-HtrA1 Fab15H6.v4.D221 in buffer and in vitreous humor. HtrA1 (200 nM) was labeled by HtrA1 ABP (10 μM) with and without anti-HtrA1 Fab15H6.v4.D221 (3.7 to 2,700 nM) pretreatment for 30 min. The intensities of active and total HtrA1 protein bands on fluorescent gel and Western blot images, respectively, were quantified by densitometric analysis and expressed as active/total ratios. The percentage of the HtrA1 ABP signal remaining assumes maximal response is 100% (control without antibody) and the maximally inhibited response is 0%. Data were fitted to a dose–response curve (GraphPad Prism) to calculate IC_50_ values. The values ±SEM are the average of triplicates. Representative gel and Western blot images are shown above each curve.

To determine if the anti-HtrA1 Fabs were successfully inhibiting HtrA1 activity in a native environment, we constructed an HtrA1-directed activity-based probe. To generate such a probe, we first determined the consensus cleavage site of HtrA1 by N-terminomics. Specifically, we compared the amino termini of proteins found in rat vitreous humor upon incubation with HtrA1 or a catalytically inactive version of HtrA1 (HtrA1^S328A^) (14). This experiment demonstrated a preference for hydrophobic residues, such as valine and leucine, at P1 (corresponding to the position N-terminal to the cleavage site) in a variety of substrates present in the vitreous humor (Fig. 2*A*). The HtrA1 cleavage-site specificity reported by N-terminomics was corroborated in a study using a peptide library to determine preferred HtrA1 cleavage sites (15). We then designed an activity-based small-molecule probe (ABP) (Fig. 2*B*), where diphenyl phosphonate was the reactive group targeting the nucleophilic active-site serine residue. To direct the ABP reactivity against HtrA1, Val and Leu were introduced at the P1 and P2 positions. Carboxytetramethylrhodamine (TAMRA) 5,6 was incorporated through a polyethylene glycol linker as a fluorescent reporter tag. As expected, the HtrA1 ABP was reactive against HtrA1 and HtrA1-PD but did not bind the respective serine-to-alanine catalytically inactive mutants (*SI Appendix*, Fig. S2*A*). Probe-labeled HtrA1 appeared as multiple bands migrating at ∼35 to 50 kDa. The 50-kDa band matches the molecular mass of intact HtrA1 protein. The two lower–molecular-mass bands are most likely the result of HtrA1 autolytic activity (16). Preincubation of either HtrA1 or HtrA1-PD with an inhibitory anti-HtrA1 Fab15H6.v4.D221 decreased active site labeling with the HtrA1 ABP in a concentration-dependent manner (Fig. 2*C*). To determine the selectivity of the ABP toward HtrA1 in a complex biological matrix, recombinant HtrA1 was added to rabbit vitreous humor followed by labeling with either fluorophosphonate (fluoroFP), a generic serine hydrolase ABP (14), or the HtrA1 ABP. While fluoroFP labeled many bands in vitreous humor, corresponding to vitreal, endogenous serine hydrolases including HtrA1, the HtrA1 ABP was more selective for HtrA1, and labeling could be inhibited by preincubation with anti-HtrA1 immunoglobulin (IgG) 15H6.v2 (Fig. 2*D*; evaluation of anti-HtrA1 specificity is described in Fig. 1 and *SI Appendix*, Fig. S1*B*). A higher–molecular-mass protein at 60 kDa, likely an unrelated vitreal serine hydrolase, was also labeled by fluoroFP and to a lesser extent by the HtrA1 ABP. However, probe labeling of this nonspecific protein was unaffected by the presence of anti-HtrA1 IgG15H6.v2. The HtrA1 ABP served as a valuable tool for tracking HtrA1 activity and determining the inhibitory potency of anti-HtrA1. We used the HtrA1 ABP in a competitive format to determine the ability of anti-HtrA1 to block HtrA1 labeling by the ABP. Anti-HtrA1 Fab15H6.v4.D221 inhibited ABP labeling in a concentration-dependent manner, both in buffer and in vitreous humor, with IC_50_ values (concentration required to block 50% of activity) of 35.9 and 51.0 nM, respectively (Fig. 2*E*). The difference in IC_50_ values likely reflects the additional contribution of endogenous HtrA1 in the vitreous humor. These data demonstrate that anti-HtrA1 Fab15H6.v4.D221 acts as a selective and potent inhibitor of HtrA1 activity. Hence, in this work, we have successfully developed an ABP with improved selectivity for HtrA1 in vitreous humor, a biologically relevant matrix, and applied the HtrA1 ABP for detecting changes in HtrA1 activity to study the pharmacodynamics of anti-HtrA1.

### ABP Detects In Vivo Inhibition of Endogenous Ocular HtrA1 Activity by Anti-HtrA1.

**Fig. 3.**
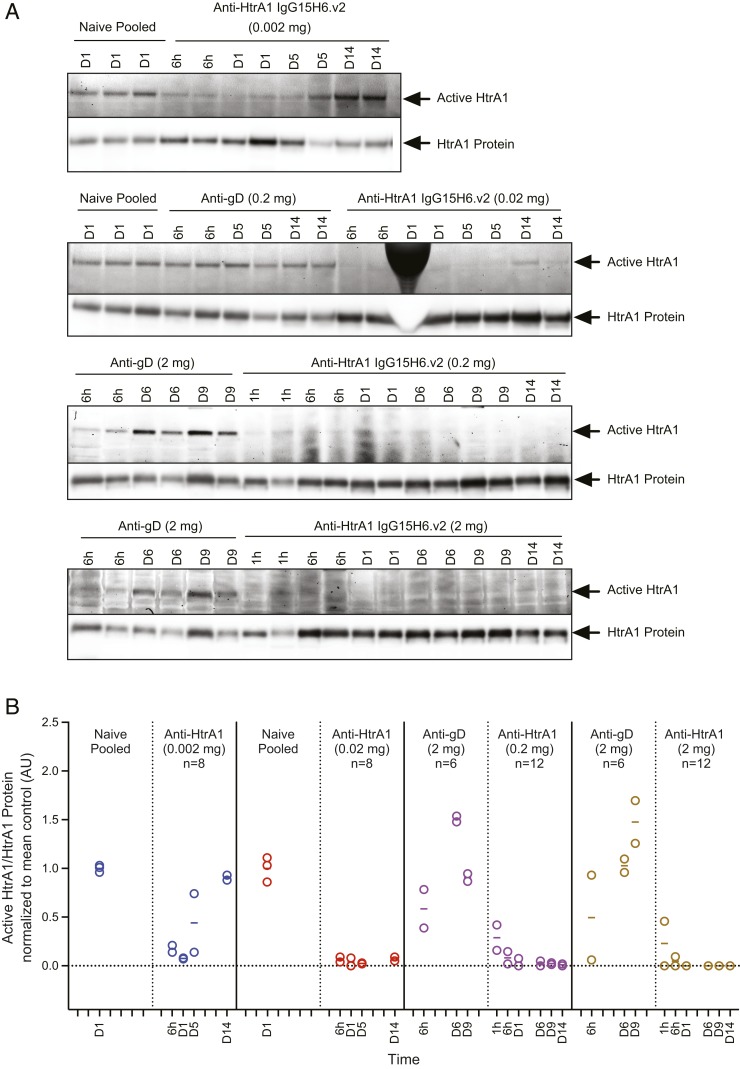
In vivo ABP labeling demonstrates activity of vitreous HtrA1 and inhibition by anti-HtrA1 (IgG15H6.v2) in rabbits. (*A*) Fluorescent gel images and Western blots for in vivo rabbit studies are shown. Arrows indicate active HtrA1 or total HtrA1 protein bands, respectively. Gel densitometric analysis of active and total HtrA1 bands was performed and expressed as ratios of active/total HtrA1 protein. Ratios were normalized to the mean value for the naïve pool or anti-gD controls that were analyzed on the same fluorescent gel image or blot. (*B*) Anti-HtrA1 IgG15H6.v2 was administered by ITV injection in rabbits followed by HtrA1 ABP labeling of harvested vitreous humor at different terminal time points post dose. The plot depicts densitometric quantification of active/total HtrA1 for each animal at terminal time points grouped by dose. Each data point represents an individual animal (with the exception of naïve pooled samples). Horizontal bars correspond to the average measurement per time point. Controls used for normalization are shown adjacent to their respective treatment groups. Solid vertical lines separate samples analyzed on different gels and blots. HtrA1 ABP labeling was completely blocked following anti-HtrA1 IgG15H6.v2 administration at 0.02, 0.2, and 2 mg per eye for at least 14 d. At the lowest dose (0.002 mg per eye) administered, HtrA1 activity rebounded to near-control levels on day 14.

To determine HtrA1 activity and inhibition in the eye in vivo following intravitreal (ITV) administration of anti-HtrA1 IgG15H6.v2 in rabbits, vitreous humor was harvested and labeled with HtrA1 ABP at different time points post dose ex vivo. HtrA1 activity could be readily detected in the vitreous humor from rabbits injected with a control Ab (anti-gD IgG1). Similar to the in vitro ABP experiments, HtrA1 ABP labeling was blocked following anti-HtrA1 IgG15H6.v2 administration at 0.02, 0.2, and 2 mg per eye for at least 14 d (Fig. 3). At 0.002 mg per eye, vitreal HtrA1 was inhibited through day 1 followed by recovery to near-baseline level by day 14. This transient inhibition at the lowest dose is consistent with reduced anti-HtrA1 IgG15H6.v2 levels over time due to clearing out of the vitreal compartment.

**Fig. 4.**
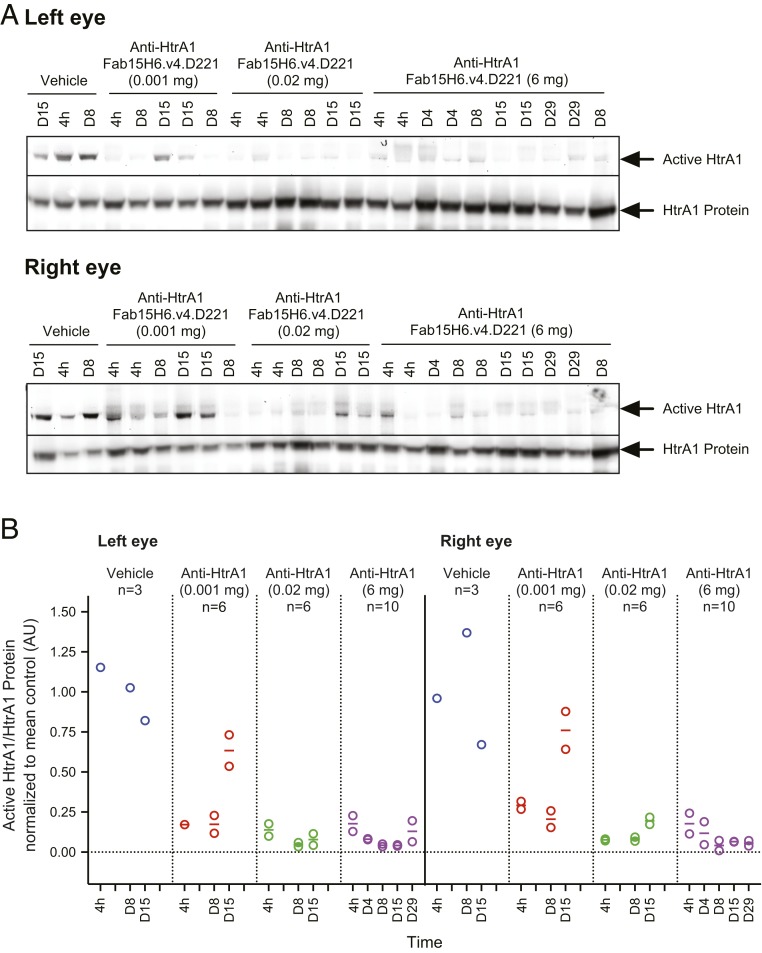
In vivo ABP labeling demonstrates activity of vitreous HtrA1 and inhibition by anti-HtrA1 (Fab15H6.v4.D221) in monkeys. (*A*) Fluorescent gel images and Western blots for in vivo monkey studies are shown. Arrows indicate active HtrA1 or total HtrA1 protein bands, respectively. Densitometric quantification was performed as described above. Active/total HtrA1 protein ratios were normalized to mean value for vehicle controls. Left-eye day 1 sample was not analyzed as the sample was lost during transport. (*B*) Anti-HtrA1 Fab15H6.v4.D221 blocked endogenous vitreal HtrA1 activity in cynomolgus monkeys in a dose-related manner while HtrA1 activity was readily detectable in animals treated with vehicle control. Inhibition was sustained into day 29 for the 6 mg per eye dose groups while the 0.001 mg per eye dose group recovered to near-control levels on day 15.

Next, we determined inhibition of HtrA1 in cynomolgus monkeys. Anti-HtrA1 Fab15H6.v4.D221 blocked vitreal HtrA1 activity in a dose-related manner while HtrA1 activity was readily detectable in animals treated with vehicle control. Transient inhibition was detected with a dose of 0.001 mg per eye; however, at the higher ITV dose ranges of 0.02 and 6 mg per eye, vitreous HtrA1 activity was completely inhibited for the duration of the study (Fig. 4). Taken together, these studies demonstrate that HtrA1 is constitutively active in the rabbit and cynomolgus vitreous humor, and that an HtrA1-directed ABP can be used to establish in vivo pharmacodynamics of the anti-HtrA1 antibody injected intravitreally.

While the HtrA1 ABP was a useful tool for monitoring HtrA1 activity and inhibition in vitreous humor, vitreous humor is not routinely collected for biomarker sampling in human clinical trials. On the other hand, aqueous humor, from the anterior chamber of the eye, can be readily collected in clinical trials, and biomarkers in aqueous humor can reflect changes occurring in the vitreous. However, using the ABP, we found that HtrA1 protease activity was not detectable in aqueous humor of preclinical animal models. As an alternative approach to discover aqueous humor biomarkers of HtrA1 protease activity, we sought to identify HtrA1 substrates that are processed by HtrA1 enzyme in the vitreous and drain to the aqueous compartment (17).

### Identification of HtrA1 Ocular Substrates by Terminal Amine Isotopic Labeling of Substrates.

**Fig. 5.**
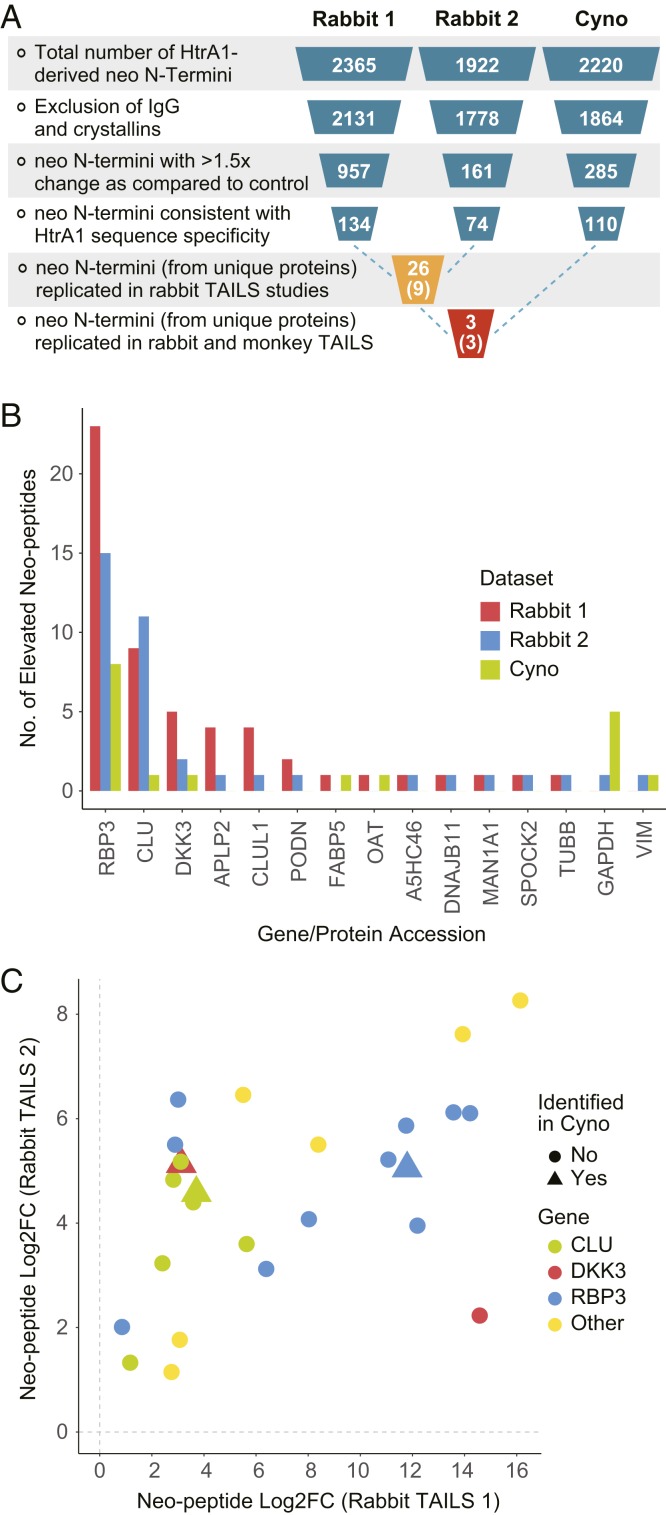
Data analysis and triaging of TAILS results. (*A*) Neo N termini were identified using TAILS and the data were processed as follows: Peptides derived from IgGs and crystallins were excluded and a cutoff of 1.5-fold (log_2_ fold change [FC] > 0.58) was applied to identify peptides that were enriched in the HtrA1-active as compared with HtrA1-inhibited samples. neo–N-terminal peptides that were not consistent with HtrA1 cleavage-site specificity, for instance, cleavages C-terminal of a basic amino acid, were excluded. From the replicate rabbit studies, we identified 26 peptides with new N termini that were derived from nine proteins. (*B*) The following proteins were identified based on their neo–N-terminal peptides replicating in the two rabbit studies: RBP3, CLU, DKK3, CLUL1, and APLP2. (*C*) From the five putative HtrA1 substrates identified in rabbit, only three matching proteins were identified in the TAILS experiment performed in monkey, namely RBP3, CLU, and DKK3. Neo–N-terminal peptides from these three proteins were consistently up-regulated in HtrA1-active as compared with HtrA1-inhibited samples, with similar fold-change magnitude.

To identify ocular substrates for HtrA1 within the enzyme’s native environment, we performed terminal amine isotopic labeling of substrates (TAILS) (18) using vitreous humor harvested from rabbits or cynomolgus monkeys treated with anti-HtrA1 antibody (HtrA1-inhibited) vs. control treatment (HtrA1-active) (*SI Appendix*, Fig. S3). The active/inhibited catalytic status of vitreal HtrA1 was further confirmed by activity-based probe labeling. We identified 2,365, 1,922, and 2,220 neo–N-terminal peptides in the rabbit (two) and cynomolgus monkey (one) studies, respectively. We then employed a filtering scheme (Fig. 5*A*) in which peptides derived from IgGs and crystallins, the two common and highly abundant ocular proteins, were excluded. We further applied a cutoff of 1.5-fold (log_2_ fold change > 0.58) to select for peptides that were robustly enriched in the HtrA1-active as compared with HtrA1-inhibited samples. We include the data for peptides identified across all species and those derived from rabbits in Dataset S1 and *SI Appendix*, Table S1, respectively. Finally, we excluded neo–N-terminal peptides that were not consistent with the HtrA1 consensus cleavage site, which is characterized by a hydrophobic amino acid at P1. From the replicate rabbit studies, we identified 26 neo–N-terminal peptides derived from nine proteins. Cumulatively, the highest number of neo–N-terminal peptides that replicated in the two rabbit studies mapped to five proteins: retinol-binding protein 3 (RBP3), Dickkopf-related protein 3 (DKK3), clusterin (CLU), CLU-like protein 1 (CLUL1), and amyloid-beta precursor-like protein 2 (APLP2) (Fig. 5*B*). From these five putative HtrA1 substrates identified in rabbits, only three matching proteins were identified in the TAILS experiment performed on samples from cynomolgus monkey, namely CLU, RBP3, and DKK3. Neo–N-terminal peptides from these three proteins were consistently up-regulated in HtrA1-active as compared with HtrA1-inhibited samples, with similar fold-change magnitude (Fig. 5*C*). Analysis of the neo–N-terminal peptide sequence suggested that RBP3 is cleaved at multiple sites, while CLU and DKK3 cleavages are mapped to a few distinct locations within the protein (*SI Appendix*, Fig. S4 and Table S1).

**Fig. 6.**
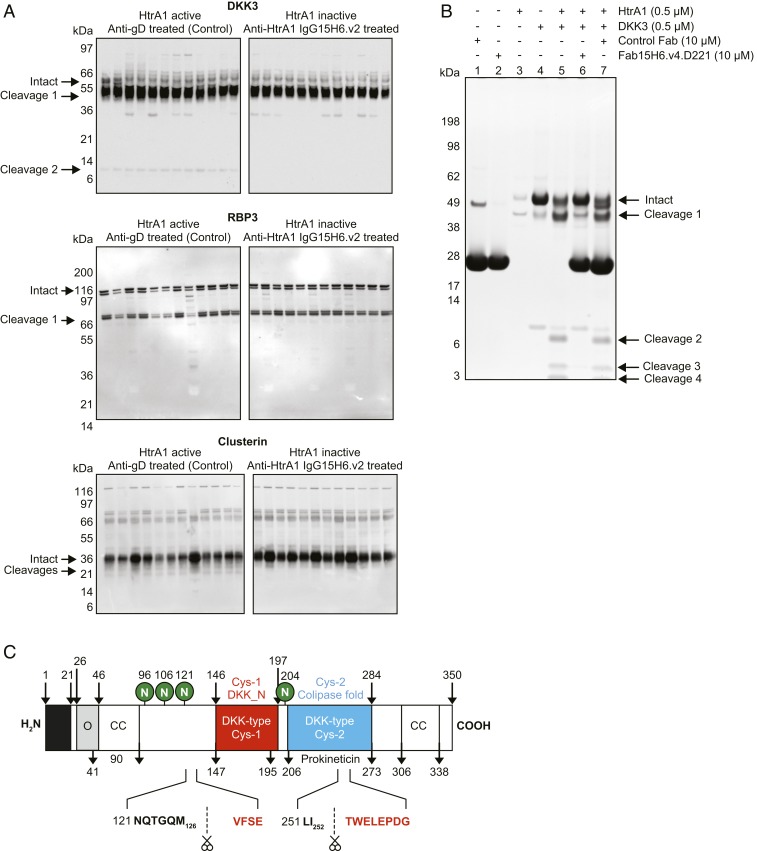
HtrA1 cleaves DKK3 at distinct sites in vitro and in vivo. (*A*) Immunoblot showing HtrA1-mediated cleavage of DKK3, RBP3, and CLU in vivo in rabbits. DKK3 cleavage products were detected in vitreous humor from individual rabbits treated with control antibody (*Left*). Upon treatment with the HtrA1-specific antibody IgG15H6.v2 (*Right*), HtrA1 protease activity was inhibited and DKK3 remained mostly intact. The masses of the cleavage fragments in vivo appear to be consistent with the two cleavage sites identified by TAILS. RBP3 and CLU cleavage fragments identified by TAILS were not readily detectable in vitreous humor. (*B*) In vitro validation of DKK3 as a substrate for HtrA1 and inhibition of cleavage by anti-HtrA1. DKK3 (4 μM) was incubated with HtrA1 (0.5 μM) in the absence or presence of anti-HtrA1 Fab15H6.v4.D221 (10 μM) or control Fab for 3 h at 37 °C. Samples were subjected to SDS/PAGE and visualized with SimplyBlue SafeStain. Lanes 1, 2, 3, and 4 correspond to control Fab, Fab15H6.v4.D221, HtrA1, or DKK3, respectively. Lane 5, 6, and 7 correspond to DKK3 + HtrA1, DKK3 + HtrA1 with addition of Fab15H6.v4.D221, or control Fab. Cleavages 1 and 2 are similar to cleavage products observed in in vivo rabbit studies as shown in *A*. DKK3 cleavage by HtrA1 was reduced (cleavage 1) or completely inhibited (cleavages 2 to 4) in the presence of anti-HtrA1 Fab15H6.v4.D221. Bands corresponding to DKK3 cleavage products were analyzed by Edman degradation. N-terminal sequences confirmed the two TAILS-predicted cleavage sites along with additional sites. (*C*) Schematic showing key domains in DKK3 and the position of validated TAILS cleavage sites.

Immunoblotting of vitreous humor collected from individual rabbits treated with anti-HtrA1 or control IgG revealed a consistent and robust in vivo cleavage of DKK3 that was absent when HtrA1 activity was blocked (Fig. 6*A*). A similar analysis was done for RBP3 and CLU. HtrA1-mediated cleavage of RBP3 was not readily detectable. CLU appeared to be cleaved when HtrA1 was active; however, the bands were not prominent, making it challenging to monitor them by immunoblotting. CLU was previously reported and characterized as a substrate for HtrA1 (19). However, in our experiments, cleavage of both CLU and RBP3 by HtrA1 was not readily or consistently detected in vitreous humor. Therefore, we focused on further characterization of DKK3. In vitro, recombinant DKK3 protein was readily cleaved by recombinant human HtrA1 (Fig. 6*B*). DKK3 cleavage by HtrA1 could be inhibited by incubation with anti-HtrA1 Fab15H6.v4.D221 but not control Fab. Amino acid sequencing of the in vitro cleaved products by Edman degradation confirmed the cleavage sites previously identified by TAILS in vivo. Consistent with HtrA1 P1 cleavage-site specificity, the two prominent DKK3 cleavage sites, which were identified both in vitro and by TAILS, were mapped at M126 in a region upstream of the N-terminal cysteine-rich domain, and at I252, within the second cysteine-rich domain (Fig. 6*C*). Thus, using an N-terminomics approach for discovery and confirmation by immunoblotting, we have identified endogenous HtrA1 substrates in rabbit and cynomolgus monkey ocular vitreous humor that can potentially serve as biomarkers of HtrA1 activity in the human eye. Importantly, the DKK3 cleavage product was also detected in aqueous humor in rabbits, and was blocked upon treatment with anti-HtrA1 IgG15H6.v2. Additionally, analysis of human cadaver vitreous and aqueous humor and GA patient aqueous humor revealed the presence of similar DKK3 cleavage products (*SI Appendix*, Fig. S5). Based on this robust signal in aqueous humor and our ability to monitor cleavage by immunoblotting, DKK3 was prioritized as a biomarker for the monitoring of HtrA1 protease activity in human clinical samples.

### Evaluation of DKK3 as a Pharmacodynamic Biomarker of Anti-HtrA1 Fab15H6.v4.D221 in Patients.

We conducted a phase 1 trial design evaluating the safety, tolerability, pharmacokinetics, and immunogenicity of Fab15H6.v4.D221 following single and multiple ITV administrations in patients with GA secondary to AMD. This study consisted of a single ascending-dose (SAD) stage with doses ranging from 1 to 20 mg per eye, and a multiple-dose (MD) stage, which evaluated three consecutive doses of 20 mg per eye every 4 wk. The SAD and MD stages of this study included mandatory serial aqueous humor sampling to allow evaluation of DKK3 cleavage as an indicator of HtrA1 protease activity upon treatment with anti-HtrA1 Fab15H6.v4.D221.

**Fig. 7.**
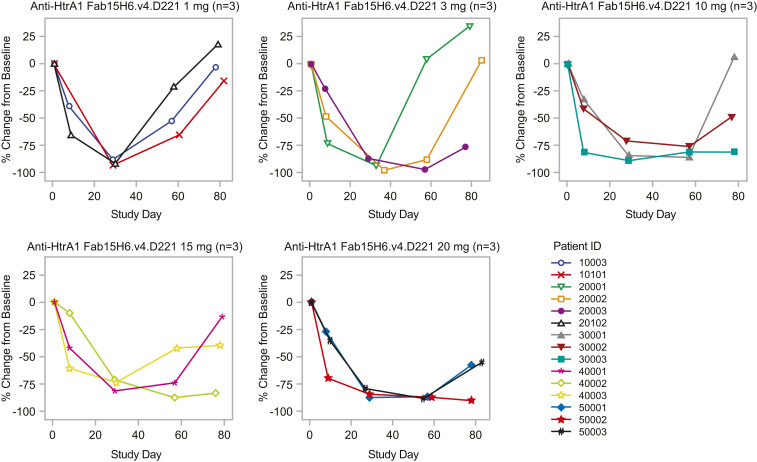
DKK3 cleavage utilization as PD biomarker in the phase 1 study following treatment with anti-HtrA1 Fab15H6.v4.D221. The SAD stage consisted of doses of anti-HtrA1 Fab15H6.v4.D221 ranging from 1 to 20 mg (*n* = 15). Levels of cleaved DKK3 were assessed by Western blot in aqueous humor of patients at baseline and at multiple time points following anti-HtrA1 treatment as a biomarker of anti-HtrA1 modulation of HtrA1 protease activity. A plot of percent change from baseline for aqueous humor cleaved DKK3 by study day is shown for each treatment group.

Aqueous humor samples from the study patients were taken at baseline and at multiple time points following anti-HtrA1 treatment. Western blot analysis revealed dose-dependent inhibition of DKK3 cleavage by Fab15H6.v4.D221 (*SI Appendix*, Fig. S6 and Table S2). Importantly, higher doses yielded longer durations of anti-HtrA1 blocking activity and, at the 20-mg dose, inhibition of DKK3 cleavage was sustained for >8 wk after a single ITV administration (Fig. 7). These results provide clear evidence of sustained pharmacological activity of Fab15H6.v4.D221 and an important framework for the design of clinical studies to test the therapeutic hypothesis that inhibition of HtrA1 will slow the progression of GA.

## Discussion

Herein, we describe the development of a potential clinical anti-HtrA1 Fab for the treatment of AMD, as well as the discovery of an HtrA1-specific pharmacodynamic biomarker and its application to clinical studies. This work demonstrates different aspects of the translational work from bench to bedside and highlights the importance of a clinical readout of pharmacological activity, within the ocular compartment, in guiding dose and dose regimen selection.

We set out to find such a biomarker by identifying substrates of the protease HtrA1, hypothesized to play a role in GA progression. Since proteases can cleave substrates promiscuously, a key to understanding the biological function of HtrA1 and its potential involvement in AMD pathology is through the identification of its endogenous substrates within the native ocular environment. While several substrates were reported for HtrA1 previously, these were identified in in vitro in-cell systems or by artificially overexpressing HtrA1. Substrates identified in these studies may therefore be separated spatially and temporally from HtrA1-active niches since their relevance for the native ocular environment has not been established.

Ocular matrices that are most readily accessible for identifying biomarkers in vivo are tears, and ocular surface tissues such as the conjunctiva and cornea. However, while studying retinal diseases such as GA, the aqueous humor and vitreous are more suitable environments for the identification and surveillance of relevant biomarkers (20). Considering this, we proceeded to identify in vivo HtrA1 biomarkers by administering a highly specific Fab directly into the vitreous humor in preclinical animal models, and demonstrated that this antibody robustly inhibited the catalytic activity of HtrA1 within its native ocular environment.

It is well-established that ITV administration of a Fab results in high exposure of ocular compartments (with minimal systemic exposure), and thus it is reasonable to conclude that the HtrA1 substrates identified in this study are derived from local rather than systemic proteolysis of the substrate. Standard proteomic approaches are subject to interexperimental variability and are very challenging to perform in matrices with a wide range of protein concentrations. To circumvent these limitations, we employed TAILS, an N-terminomic approach that enriches N-terminal peptides. To increase confidence in our results, we repeated this experiment three times in two different species that closely mimic the human ocular physiology. The vitreous humor is a complex matrix to work with, as it contains several highly abundant proteins such as albumin, globulins, coagulation proteins, and complement factors that have accumulated from local secretion or diffusion from surrounding tissue and vasculature (20). This made TAILS analysis challenging and required several data-filtration steps to identify distinct substrates specific for HtrA1. The only three endogenous substrates that were reproducibly found in all experiments were CLU, RBP3, and DKK3.

CLU has been previously reported as an HtrA1 substrate (19). It is the principal protein of the Apo J lipoprotein particle. The exact function of CLU has not been fully elucidated, but has been shown to be antiinflammatory, counteracting the complement membrane attack complex (21) and other proinflammatory factors, as well as a major component in drusen deposits (22). CLU expression is increased in retinitis pigmentosa (23). Similar to DKK3 and RBP3, CLU is primarily produced by Müller cells (24).

RBP3 is highly expressed in the retina and is a principal component of the photoreceptor extracellular matrix (25). RBP3 acts to shuttle all-*trans* retinol and 11-*cis* retinal between the RPE and the photoreceptor, and therefore is an essential component of the visual cycle, a process that involves the cycling of retinoids between the photoreceptor rod outer segments and the RPE (2). This process is not only vital to visual phototransduction but also important for the viability of the photoreceptors. By removing all-*trans* retinol from rod outer segments, RBP3 prevents the formation of lipofuscin, a toxic by-product of the visual cycle (26). In addition, it has antioxidant properties that protect the photoreceptors (27). HtrA1 cleaves RBP3 at multiple sites, likely inactivating the protein. Interestingly, loss-of-function mutations in RBP3 result in retinal degeneration associated with retinitis pigmentosa (28). Although RBP3 and CLU were validated as HtrA1 substrates, their cleavage fragments were not reliably detectable by immunoblotting in the vitreous, thus making them unsuitable clinical biomarkers. We therefore decided to focus on DKK3 as the biomarker of choice for further clinical investigation. The intact DKK3 form as well as its HtrA1-specific cleavage products were readily detectable in the aqueous humor of human cadavers and GA patients, raising the possibility that DKK3 may be a suitable biomarker for anti-HtrA1 therapy in the clinical setting.

DKK proteins are modulators of the Wnt pathway. While DKK1 and DKK2 were shown to bind the Wnt coreceptors LRP5/6 and inhibit canonical Wnt signaling, DKK3 does not bind LRP5/6. The effect of DKK3 on Wnt signaling could nevertheless be dependent on the specific cellular environment. Recently, DKK3 was reported to bind receptors on endothelial cells and induce migration in these cells (29). However, we were not able to reproduce earlier findings that DKK3 affects the migration of vascular endothelial cells. Various in vivo studies have reported on effects of DKK3 on atherosclerosis development (30). Hence, it is possible that HtrA1 affects vascular functions through DKK3 in the eye, though we have not been able to confirm this function in vitro (31). Further studies are needed to determine how HtrA1-mediated proteolysis of DKK3, RBP3, and CLU may relate to genetic variation in the HtrA1/ARMS2 region and the risk for AMD and the progression of established AMD.

Several vitreous biomarkers for AMD have previously been reported, including proteins such as vascular endothelial growth factor (32), opticin (33), and vitronectin (34), as well as several intravitreal RNAs (35). To our knowledge, DKK3 is unique in this context and serves as a potential protease-mediated biomarker for an intravitreal study.

This study also demonstrates that HtrA1 is proteolytically active under homeostatic conditions in the eye. One of the nuances of biomarker discovery is that even if one is to demonstrate robustness of a biomarker in an in vitro or preclinical model, it may not be equally robust in human clinical samples. In the case of biomarkers derived from proteolysis events, such poor translatability could be due to lack of protein sequence homology, protein conformity irregularities, or differences in stability of proteolytic fragments. Here we demonstrated that after administration of an anti-HtrA1 antibody in the eye of patients with GA, cleaved DKK3 was detected at decreased levels in the aqueous humor. This result in patients recapitulated the pharmacodynamic properties observed in our preclinical systems, validating DKK3 as a robust pharmacodynamic biomarker for monitoring inhibition of HtrA1 in the treatment of ocular disease by intravitreal injection.

## Materials and Methods

### Construction, Expression, and Purification of Recombinant Human HtrA Proteins.

Human full-length HtrA1 (HtrA1) and its catalytically inactive form HtrA1(S/A), in which Ser328 was mutated to Ala, were expressed in insect cells and purified as described. The human HtrA1 protease domain (HtrA1-PD) and its catalytically inactive form [HtrA1-PD(S/A)] as well as the N domain-deleted HtrA1 (HtrA1-PD/PDZ) were expressed in *E. coli* and purified as described (15). Murine protease domain (muHtrA1-PD) was expressed in *E. coli* and purified as described (8). Detailed description in *SI Appendix*.

### Generation of Anti-HtrA1 Antibodies and Determination of Binding Affinity and Specificity of Anti-HtrA1 Fabs.

Description in *SI Appendix*.

### Designing, Building, and Characterizing an HtrA1-Specific Activity-Based Profiling Probe.

#### Determining the proteolytic cleavage specificity of HtrA1.

Rat vitreous samples were collected from Sprague–Dawley females by removing the vitreal sac after euthanasia by CO_2_ and placing eight vitreal sacs at a time on a 0.45-µm SpinX filter (Corning) wetted with 20 µL 50 mM Tris (pH 8.0), 200 mM NaCl, 3× cOmplete Protease Inhibitor Mixture (Roche), and then spinning down fluid for 15 min at 12,000 rpm at 4 °C. Fluid was pooled from 150 rats and protein concentration was measured by BCA (Thermo Fisher Scientific) and stored at −80 °C. After thawing on ice, 773 µM mouse HtrA1 or HtrA1(S/A) was added to 1.5 mL vitreal fluid for 3 h at 37 °C and then subjected to an N-terminomic approach as previously described (36). Peptide sequences identified using this methodology were analyzed using iceLogo (37).

#### Generation of an HtrA1-specific activity-based profiling probe.

The ActivX serine hydrolase probe (fluoroFP general probe) was purchased from Thermo Fisher Scientific (88318). The specific HtrA1 TAMRA ABP (HtrA1 ABP) was synthesized by WuXi AppTec. Synthesis of ABPs with a diphenylphosphonate reactive group has previously been described by Pan et al. (38). Specific details of the synthesis scheme for the HtrA1 ABP are provided in *SI Appendix*, *Methods*. The final product was assessed by LC-MS and NMR (*SI Appendix*, Fig. S2 *B*–*D*) and had a high degree of purity (>87%). The stereochemistry at the carbon with the isopropyl substituent was unknown and assigned randomly as diastereomer 1 or 2. The active compound was determined experimentally by the compound’s ability to covalently label recombinant HtrA1 protein.

#### Validation of an HtrA1-specific activity-based profiling probe.

Activity labeling experiments were performed at room temperature protected from light. HtrA1 and HtrA1-PD and their respective catalytically inactive S/A mutants HtrA1(S/A) and HtrA1-PD(S/A) (360 nM final) were incubated with HtrA1 ABP (10 µM final) in PBS for 1 h. To assess the inhibition of HtrA1 proteolytic activity by anti-HtrA1 Fab15H6.v4.D221, HtrA1 (400 nM) or HtrA1-PD (200 nM) was preincubated with different concentrations of anti-HtrA1 Fab15H6.v4.D221 for 30 min prior to addition of HtrA1 ABP (10 µM final).

A similar protocol was applied to monitor inhibition in a complex proteome such as vitreous humor using either the ActivX TAMRA-FP (fluoroFP) general serine hydrolase ABP (Thermo Fisher Scientific; 88318) or HtrA1 ABP. For experiments with rabbit vitreous humor, HtrA1 (150 nM) was preincubated for 1 h with increasing concentrations of anti-HtrA1 IgG15H6.v2 (0.01, 0.1, or 1 µM) in PBS containing 20% rabbit vitreous humor before adding either probe for 1 h. Reactions were heated in SDS/PAGE sample buffer at 70 °C for 10 min to stop labeling and then separated on 8, 10, or 4 to 12% Bis-Tris gels (Thermo Fisher Scientific). TAMRA fluorescence in the gel was scanned on a Typhoon TRIO variable-mode imager (GE Healthcare Life Sciences) with Cy3 filters and green (532-nm) laser (excitation 552 nm, emission 578 nm). The same gels used for fluorescent gel imaging were blotted onto a nitrocellulose membrane using the iBlot Transfer System (Thermo Fisher Scientific) and total HtrA1 protein levels were detected by standard Western blot as described. Protein bands on fluorescent gels or blots were quantified by densitometry using ImageQuant TL software (GE Biosciences).

#### HtrA1-specific activity-based profiling probe-based competition assay to determine IC_50_ values.

Human recombinant HtrA1 (200 nM) in assay buffer (50 mM Tris⋅HCl, pH 8.0, 200 mM NaCl, 0.25% CHAPS) or in assay buffer containing 20% rabbit vitreous humor was preincubated with and without anti-HtrA1 Fab15H6.v4.D221 at the indicated concentrations (3.7 to 2,700 nM) for 30 min to label with HtrA1 ABP (10 μM) in the dark for 1 h at room temperature, terminated in SDS/PAGE sample buffer, separated by SDS/PAGE, and visualized by in-gel fluorescence scanning followed by standard Western blotting as described. The activity remaining was determined by densitometric quantification of the ratio of probe-labeled HtrA1 protein to total HtrA1 protein levels. The percentage activity was calculated by normalizing the response to run between 0 and 100% with the maximum response defined as 100% and the minimum response defined as 0% for the buffer and vitreous conditions. IC_50_ values were determined by fitting a dose–response curve using GraphPad Prism software.

### Animal Studies to Further Characterize the HtrA1-Specific Activity-Based Profiling Probe.

Animals were treated and handled in accordance with the Animal Welfare Act, *Guide for the Care and Use of Laboratory Animals* (39) with protocols approved by Genentech, Inc., Institutional Animal Care and Use Committee, and Association for Research in Vision and Ophthalmology Statement for the Use of Animals in Ophthalmic and Vision Research.

#### In vivo rabbit studies.

For rabbit pharmacokinetic/pharmacodynamic studies and vitreous substrate identification by TAILS, Fab15H6.v2 was reformatted to human IgG1, expressed in Chinese hamster ovarian cells, and purified by protein A chromatography. The determined endotoxin level of the preparation was 0.025 endotoxin unit (EU) per milligram protein. Dutch belted rabbits (Covance Research Products) were at least 4 mo of age and ranged in weight from 1.5 to 2.5 kg at study initiation. Each animal was housed individually with species-specific standard certified commercial chow and water ad libitum, along with supplemental dietary enrichment.

Intravitreal injections and ophthalmic examinations were performed by a board-certified veterinary ophthalmologist. Topicamide (1%) and/or phenylephrine (two drops) were applied to each eye for full pupil dilation. Prior to the ITV injections, animals were anesthetized with intramuscular injections of xylazine (5 mg/kg), followed by ketamine (22 mg/kg) 10 min apart. A topical anesthetic (e.g., 1% proparacaine) was instilled in each eye before the dose administration. A wire speculum was used to retract the eyelids. To minimize external ocular irritation, eye preparation was limited to a dose site-specific cleaning with dilute 1% povidone iodine solution (prepared with sterile saline and 5% povidone iodine) and rinsed with sterile saline prior to the dose administration. Doses were administered by ITV injection, 50 µL per eye. A sterilized 100-µL Hamilton Luer lock syringe with a 30-gauge, 1/2-inch needle attached was used for each dose administration. Syringes were filled with 50 µL of IgG15H6.v2 under a laminar flow hood immediately before dosing in each eye. A topical antibiotic (gentamicin) was applied to each eye following dosing at veterinary discretion. After injection, rabbits were monitored twice daily and a sustained-release opioid (buprenorphine) was administered twice a day for at least 3 d on study. Animals were examined by veterinary staff prior to discontinuation of pain control. At predetermined times, animals were anesthetized using sodium pentobarbital and exsanguinated.

For the pharmacokinetic/pharmacodynamic studies, 50 naïve Dutch belted rabbits were randomly assigned to the following groups, and given a single-dose administration of control antibody or anti-HtrA1 IgG15H6.v2 (endotoxin levels 0.025 EU/mg protein) by bilateral intravitreal injection at the following doses: naïve (*n* = 4), anti-glycoprotein IgG1 (anti-gD; endotoxin levels 0.013 EU/mg protein) 2 mg per eye (*n* = 6), anti-HtrA1 IgG15H6.v2 2 mg per eye (*n* = 12), anti-HtrA1 IgG15H6.v2 0.2 mg per eye (*n* = 12), anti-HtrA1 IgG15H6.v2 0.02 mg per eye (*n* = 8), and anti-HtrA1 IgG15H6.v2 0.002 mg per eye (*n* = 8). Vitreous humor was harvested from euthanized animals for pharmacodynamic analysis at 1 h, 6 h, and 1, 6, 9, and 14 d post dosing (*n* = 2 per time point).

For the TAILS studies, 20 naïve Dutch belted rabbits were randomly assigned to either human anti-gD (control; endotoxin levels 0.013 EU/mg protein) or anti-HtrA1 IgG15H6.v2 (endotoxin levels 0.025 EU/mg protein) in replicate studies (*n* = 8 and *n* = 12 for rabbit experiments 1 and 2, respectively). The dose was selected based on results of dose-ranging studies indicating that 0.2 mg per eye was necessary to achieve complete and sustained inhibition of HtrA1 protease activity through day 14. Vitreous was collected on day 14 and processed for activity analysis and TAILS experiment. Vitreous humor (0.1 mL) was labeled with an HtrA1-specific activity probe (10 µM) for 1 h at room temperature and analyzed by fluorescent gel imaging and Western blot analysis, as described. After removing 0.1 mL of vitreous humor for activity probe labeling studies, protease inhibitor mixture (cOmplete Ultra EDTA-free tablets; Roche; 05892791001) was added to the remaining vitreous humor and stored at −80 °C until TAILS analysis.

#### In vivo cynomolgus monkey studies.

Cynomolgus monkey studies were performed at Charles River Laboratories Montreal. Drug-naïve cynomolgus monkeys (RMS Houston) ranging from 2 to 3.5 y of age and from 2.5- to 3.5-kg weight were used. Animals were allowed to acclimate for a minimum of 4 wk prior to the start of treatment and were group-housed (up to three animals per cage) in stainless steel mesh-floor cages. PMI Nutrition International Certified Primate Chow no. 5048 and fresh water (ad libitum) were provided. Environmental controls were set to maintain a temperature of 20 to −26 °C, a relative humidity of 30 to −70%, and a 12-h light/12-h dark cycle. Psychological and environmental enrichment was provided to animals as per standard operating procedures.

A topical antibiotic (tobramycin ophthalmic drops) was applied twice on the day before and the day following each injection. Prior to dosing, animals received an intramuscular injection of a sedative mixture of ketamine (5 mg/kg) and dexmedetomidine (0.01 mg/kg) followed by an isoflurane/oxygen mix through a mask as necessary to maintain anesthesia. Following completion of the dosing procedure (as considered necessary), animals received an intramuscular injection of 0.1 mg/kg atipamezole, a reversal agent for dexmedetomidine. The conjunctivae were flushed with benzalkonium chloride (Zephiran) diluted in sterile water, U.S.P. to 1:10,000 (vol/vol). Mydriatic drops (1% tropicamide and/or 2.5% phenylephrine) were applied as needed. A topical anesthetic (e.g., 0.5% proparacaine) was instilled before dose administration. Dose formulations were administered bilaterally by intravitreal injection on day 1; a 1-mL syringe and a 30-gauge, 1/2-inch needle was used. The dose volume was 50 µL per eye. In order to mimic clinical dosing, eyes were dosed in the inferotemporal quadrants, that is, in 5 and 7 o’clock positions for the left and right eyes, respectively (when facing the animal). Intravitreal injections were performed by a board-certified veterinary ophthalmologist. A topical antibiotic (tobramycin ophthalmic ointment) was applied after dose administration. Eyes were examined by slit-lamp biomicroscopy and/or direct and indirect ophthalmoscopy following completion of each treatment to confirm the location and appearance of the dose and document any abnormalities (especially to the lens, vitreous, and retina) caused by the administration procedure.

Animals surviving until scheduled euthanasia were fasted overnight before their scheduled necropsy. Prior to necropsy, a sedative (ketamine HCl for injection, U.S.P.) was administered by intramuscular injection. Animals underwent exsanguination by incision of the axillary or femoral arteries following anesthesia by intravenous injection of sodium pentobarbital.

Twenty-five male cynomolgus monkeys were divided into four groups and given a single-dose administration of vehicle (*n* = 3) or Fab15H6.v4.D221 (endotoxin level 0.0015 EU/mg protein) by bilateral ITV injection at the following doses: 0.001 mg per eye (*n* = 6), 0.02 mg per eye (*n* = 6), and 6 mg per eye (*n* = 10). Pharmacodynamic analysis was performed on vitreous humor collected from the left and right eyes of cynomolgus monkeys at 4 h and on days 4, 8, 15, and 29. A vitreous humor sample from the 0.001 mg per eye (left eye) dose group was lost during transport and not included in the analysis.

The HtrA1 ABP was added at a final concentration of 10 μM in 150 μL of vitreous humor and incubated at room temperature for 1 h. Immunoprecipitation of HtrA1 enriched the detection of the HtrA1 ABP-labeled HtrA1 signal in vitreous humor. For the HtrA1 immunoprecipitation, magnetic Dynabeads protein G (Thermo Fisher Scientific) was incubated with human anti-HtrA1 antibody (anti-HtrA1:7027 6G5; Genentech) for 1 h prior to washing and then incubated with HtrA1 ABP-labeled vitreous humor overnight at 4 °C. Elution in SDS/PAGE sample buffer was performed by heating the beads for 10 min at 70 °C. Samples were analyzed by fluorescent gel imaging and Western blot analysis, as described. For TAILS analysis, vitreous humor was pooled from anti-HtrA1 Fab15H6.v4.D221-treated animals, where HtrA1 activity was inhibited (day 15 samples from 0.02 and 6 mg per eye dose groups) and all vehicle-treated samples served as control.

#### Ocular tissue and fluid isolation and processing.

Ocular tissue and fluids were collected from each animal post dose at prespecified terminal time points. The eyes (euthanized animals only) were enucleated from the animals and the aqueous humor (∼0.05 to 0.1 mL), vitreous humor (∼1.0 mL), and retina were collected from both eyes of each animal according to standard procedures. Briefly, aqueous humor was collected first, using a syringe/needle. The eye was then dissected to remove the cornea, iris, and lens, prior to collecting vitreous humor using a pipette. The sclera was cut in sections in order to flatten the globe for collection of the retina using forceps, without removing the pigmented epithelium. Tissues and fluids were snap-frozen using liquid nitrogen, placed on dry ice, and stored at −80 °C until pharmacodynamic analysis.

Fresh vitreous humor (rabbits) or vitreous humor thawed on ice (monkeys) was transferred into 2-mL bead-beating tubes prefilled with 2.4-mm metal beads (101058-604; VWR International) and processed as follows. The vitreous humor was homogenized using the “soft tissue” program (5,800 rpm for 2 × 15 s with a pause of 30 s) on the Precellys Evolution homogenizer (Bertin Technologies). The vitreous homogenate was centrifuged at 5,000 × *g* for 2 min at 4 °C in a 5424R centrifuge (Eppendorf) and the supernatant was transferred into new tubes and stored at −80 °C until pharmacodynamic analysis. The aqueous humor was stored at −80 °C, thawed on ice, and analyzed directly with no processing steps required.

For rabbit and cynomolgus monkey pharmacodynamic studies, quantitative analysis of relevant HtrA1 band densities was carried out on ABP fluorescent gel images and Western blots and expressed as a ratio of active HtrA to total HtrA1 protein. The calculated active/total HtrA1 ratios were normalized to either naïve, vehicle, or IgG controls that were separated on the same gels and blots. Active/total ratios are meaningful for intraexperiment analysis but not interexperiment analysis due to potential variability in the quantification processes of different gels and blots.

#### Western blot analysis.

Samples were separated on Bis-Tris gels (Thermo Fisher Scientific) and transferred to nitrocellulose membranes by electroblotting with the iBlot Transfer System (Thermo Fisher Scientific). Membranes were blocked with 3% milk in PBS-Tween (PBS with 0.05% [vol/vol] Tween 20) for 1 h, washed with PBS-Tween, and incubated with primary antibodies (0.1 µg/mL) prepared in 2.5% BSA in PBS-Tween overnight at 4 °C. Primary antibodies used include anti-DKK3 biotinylated polyclonal goat antibody (R&D Systems; BAF1118), anti-RBP3 rabbit polyclonal antibody (Proteintech; 14352-1-AP), anti-HtrA1:7816 19G10 mouse IgG2a monoclonal antibody (Genentech), and human CLU biotinylated goat polyclonal antibody (R&D Systems; BAF2937). Anti-RBP3 and anti-HtrA1:7816 19G10 were biotinylated using EZ-Link Sulfo-NHS-LC-Biotin (Thermo Fisher Scientific; 21327), as specified by the manufacturer. The blots were washed with PBS-Tween and developed with HRP-coupled streptavidin (Thermo Fisher Scientific; 21140) diluted 1:20,000 in 2.5% BSA PBS-Tween. Blots were developed by a chemiluminescence method (SuperSignal West Pico Chemiluminescent Substrate; Thermo Fisher Scientific; 34077) and scanned on an ImageQuant LAS-4000 (GE Biosciences) Imaging System. The intensities of relevant protein bands were quantified by densitometry using ImageQuant TL software (GE Biosciences).

### Identification of Proteolytic Substrates of HtrA1 in Preclinical Animal Models.

#### Terminal amine isotopic labeling of substrates.

The vitreous from the eyes of rabbits or cynomolgus monkeys treated with control antibody (anti-gD IgG1) or anti-HtrA1 IgG15H6.v2 (rabbits) or anti-HtrA1 Fab15H6.v4.D221 (monkeys) were collected followed by chloroform/methanol precipitation to remove free amines from the buffer. Sample concentration was determined using the Bradford assay and 0.2 to 0.4 mg per condition was used for N-terminal enrichment following the TAILS protocol described by Kleifeld et al. (18). Both the native and neo (post proteolysis) N termini of proteins from samples treated with either the control or anti-HtrA1 antibody were reductively methylated with heavy (^13^CD_2_O) and light (^12^CH_2_O) formaldehyde at a final concentration of 40 mM, respectively, in the presence of 20 mM sodium cyanoborohydride (NaBH_3_CN). Labeling was reversed for the second bioreplicate of the rabbit study. Reductive methylation was performed at 37 °C overnight followed by a second addition of fresh formaldehyde (20 mM) and NaBH_3_CN (10 mM) at 37 °C for 2 h. The reaction was quenched using glycine at a final concentration of 100 mM followed by chloroform/methanol precipitation to remove excess amount of reductive methylation reagent. Proteins were then subjected to tryptic digestion (enzyme:substrate ratio of 1:100) at 37 °C overnight. The newly formed N termini were captured with HPG-ALD polymer (5× excess), which was previously washed with a 15× volume of water by centrifugation at 3,000 × *g* in a Centricon with a molecular weight cutoff (MWCO) of 3 kDa. Negative selection of the dimethylated N termini was performed in the presence of 20 mM NaBH_3_CN at 37 °C overnight. Glycine (100 mM) was added to minimize nonspecific binding of peptides to the polymer. The enriched N-terminal peptides were collected by centrifugation at 10,000 relative centrifugal force using a Centricon with an MWCO of 10 kDa. The samples were acidified using 20% trifluoroacetic acid and desalted using a C_18_-stage tip followed by mass spectrometric analysis.

#### Liquid chromatography-tandem mass spectrometry analysis.

The peptide mixture was reconstituted in solvent A (2% acetonitrile [ACN]/0.1% formic acid [FA]/water) and loaded onto a C_18_ Symmetry column (1.7-μm ethylene bridged hybrid-130 [BEH-130], 0.1 × 100 mm; Waters) using a nanoAcquity ultra performance liquid chromatography (Waters) at a flow rate of 1.5 µL/min. A gradient of 2 to 25% solvent B (0.1% FA/2% water/ACN) at 1.0 µL/min was applied over 85 min with a total analysis time of 120 min to separate the peptides. Peptides were eluted directly into an Advance CaptiveSpray ionization source (Michrom BioResources/Bruker) with a spray voltage of 1.3 kV and were analyzed using an LTQ Orbitrap Elite mass spectrometer (Thermo Fisher Scientific). Precursor ions were analyzed in the Orbitrap (60,000 mass resolution, automatic gain control [AGC] target 1E6, maximum ion time 500 ms). MS/MS was performed in the LTQ with the instrument operated in data-dependent mode whereby the top 15 most abundant ions were subjected to collision-induced dissociation fragmentation (normalized collision energy [NCE] 35%, AGC 3E4, maximum ion time 100 ms). Data were also acquired on the Orbitrap Fusion Lumos instrument (Thermo Fisher Scientific), where peptides were loaded on a 25-cm capillary column (100-µm internal diameter) packed with Waters nanoAcquity M-Class 1.7-µm BEH material at a flow rate of 0.8 µL/min via a Dionex Ultimate 3000 RSLCnano Proflow System (Thermo Fisher Scientific). A gradient of 2 to 30% B was applied over a period of 140 min with a total analysis time of 185 min. Peptides were analyzed on the Orbitrap Fusion Lumos using a top-speed method to ensure an MS1 scan was taken every 1 s. Precursor ions were analyzed in the Orbitrap (120,000 mass resolution, AGC target 1E6, maximum injection time 50 ms). Fragmentation was performed using high-energy collisional dissociation (NCE 30%, AGC 2E4, maximum injection time 35 ms) and fragment ions were analyzed in the ion trap.

#### Proteomic data analysis.

MS/MS data were searched using the Mascot search algorithm (Matrix Sciences) against a concatenated forward–reverse target–decoy database (UniProtKB concat) consisting of rabbit or cynomolgus proteins and common contaminant sequences. Spectra were assigned using a precursor mass tolerance of 50 ppm and fragment ion tolerance of 0.8 Da. Static modifications included carbamidomethyl cysteine (+57.0215 Da) and dimethyl lysine (light, +28.0303 Da); variable modifications included oxidized methionine (+15.9949 Da); the following modifications at protein N termini: Ac (+42.0106 Da) and pyroGlu (−17.0266 Da); and peptide N-terminal modifications included dimethylation (light +28.0303 Da), dimethylation (heavy, +34.0615 Da), and dimethyl lysine (heavy, +6.0312 Da). Up to two miscleavages C-terminal to arginine were allowed. Peptide spectral matches (PSMs) were filtered at 5% false discovery rate (FDR) (40). A subset of PSMs containing N-terminal dimethylation was subsequently quantified at peptide level using the VISTA Quant algorithm (40, 41). A peptide start position was obtained which allowed us to distinguish true and neo N termini. A log_2_(control Ab/anti-HtrA1) threshold was set to ≥0.58 for any peptides to be considered more abundant in the control group than in the HtrA1 inhibition group. Any potential substrates of HtrA1 must be observed in both bioreplicates of the rabbit study, or in one bioreplicate of rabbit as well as in cynomolgus monkey. Mass spectrometry data was deposited into the MassIVE database (ID MSV000084831).

### In Vitro Cleavage Assay.

Human recombinant DKK3 protein (4 μM) (R&D Systems; 1118-DK) was mixed with HtrA1 (0.5 μM) in cleavage buffer (50 mM Tris⋅HCl, pH 8.0, 200 mM NaCl, 0.25% CHAPS) in the absence or presence of anti-HtrA1 Fab15H6.v4.D221 (10 μM) or control Fab (10 μM) for 3 h at 37 °C. Reactions containing only DKK3, HtrA1, anti-HtrA1 Fab15H6.v4.D221, or control Fab in cleavage buffer served as controls. Samples were subjected to SDS/PAGE and subsequent staining with SimplyBlue SafeStain (Thermo Fisher Scientific; LC6060) according to the manufacturer’s instructions. For N-terminal sequencing by Edman degradation, in vitro cleavage reactions were incubated for 24 h at 37 °C and separated by SDS/PAGE, blotted to a polyvinylidene difluoride membrane, and stained with Coomassie blue stain. Bands representing cleaved DKK3 were excised and analyzed by Edman degradation.

### Phase 1 Study and Validation of DKK3 as a Pharmacodynamic Biomarker.

Human subjects were included in this study. The study was conducted in accordance with the principles of the Declaration of Helsinki and Good Clinical Practice. Approval from a central institutional review board and ethics committee (Quorum Review IRB) was obtained prior to phase 1 study initiation at sites. Patient consent was obtained before enrollment. No animal subjects were included in this study.

This phase 1 study was an open-label trial designed to evaluate the safety, tolerability, pharmacokinetics, and immunogenicity of anti-HtrA1 Fab15H6.v4.D221, following single and multiple ITV administrations in patients with GA secondary to AMD. Anti-HtrA1 Fab15H6.v4.D221 is also called FHTR2163 or RO7171009 and was supplied by Genentech.

The study consists of a SAD stage with doses ranging from 1 mg per eye to 20 mg per eye (*n* = 15), and an MD stage with the maximum tested dose of 20 mg administered every 4 wk for three doses (*n* = 13). All participants are patients with GA. The SAD and MD stages of this study required mandatory aqueous humor collection to ensure a meaningful assessment of ocular pharmacokinetics and pharmacodynamics and to explore their relationship in aqueous humor. Exploratory pharmacodynamic biomarker DKK3 was monitored in aqueous humor by Western blot analysis to assess if the clinically achieved exposures to anti-HtrA1 Fab15H6.v4.D221 were sufficient to produce the desired effect on the intended molecular target (i.e., HtrA1).

### Materials and Data Availability.

Proprietary information or materials provided by Genentech are not available to the public. Mass spectrometry data was deposited into the MassIVE database (ID MSV000084831).

## Supplementary Material

Supplementary File
