# Supplementary material for: Development of a therapeutic anti-HtrA1 antibody and the identification of DKK3 as a pharmacodynamic biomarker in geographic atrophy
Source: Proc Natl Acad Sci U S A. 2020 Apr 28;117(18):9952–63. doi: 10.1073/pnas.1917608117 (PMC7211935; doi:10.1073/pnas.1917608117)
Supplement: Supplementary File [file pnas.1917608117.sapp.pdf]

Supplementary Information for

Development of a therapeutic anti-HtrA1 antibody and the identification of DKK3 as a pharmacodynamic biomarker in geographic atrophy

Irene Tom[1§], Victoria C. Pham[2§], Kenneth J. Katschke Jr.[3], Wei Li[4], Wei-Ching Liang[5], Johnny Gutierrez[1], Andrew Ah Young[4], Isabel Figueroa[6], Shadi Toghi Eshghi[1], ChingWei V. Lee[7], Jitendra Kanodia[8], Scott J. Snipas[9], Guy S. Salvesen[9], Phillip Lai[10], Lee Honigberg[1], Menno van Lookeren Campagne[11], Daniel Kirchhofer[4], Amos Baruch[12]*, and Jennie R. Lill[2]*

[1]OMNI Biomarker Department, Genentech Inc. South San Francisco, CA 94080

[2]Department of Microchemistry, Proteomics & Lipidomics, Genentech Inc. South San Francisco, CA 94080

[3]Department of Immunology, Genentech Inc. South San Francisco, CA 94080

[4]Department of Early Discovery Biochemistry, Genentech Inc. South San Francisco, CA 94080

[5]Department of Antibody Discovery, Genentech Inc. South San Francisco, 94080

[6]Drug Metabolism, Pharmacokinetics and Bioanalysis, Abbvie, South San Francisco, CA 94080

[7]23&Me, South San Francisco, CA 94080

[8]Theravance Biopharma, South San Francisco, CA 94080

[9]National Cancer Institute-Designated Cancer Center, Sanford-Burnham Prebys Medical Discovery Institute, La Jolla, CA 92037

[10]Early Clinical Development OMNI Department, Genentech Inc. South San Francisco, CA 94080

[11]Inflammation & Oncology Research. Amgen, 1120 Veterans Blvd, South San Francisco, CA 94080

www.pnas.org/cgi/doi/10.1073/pnas.1917608117

[12]Biomarker Development, Calico Life Sciences, South San Francisco, CA 94080

***Corresponding Authors**

Amos Baruch, Ph.D.

Biomarker Development, Translational Medicine, Calico Labs, South San Francisco, CA 94080

Email: barucha@calicolabs.com; Telephone: (650)267-7919

Jennie R. Lill, Ph.D.

Department of Microchemistry, Proteomics & Lipidomics

Genentech, 1 DNA Way, MS463A South San Francisco, CA 94080

Email: jlill@gene.com; Telephone: (650) 467-7430

§: These authors contributed equally to this work

This PDF file includes:

Supplemental Methods

Figures S1 to S6

Tables S1 & S2

Other supplementary materials for this manuscript include the following:

Dataset S1

Supplemental Methods

Construction, expression and purification of recombinant human HtrA proteins

Human HtrA2-PD/PDZ (A134-E458), HtrA3-PD/PDZ (L130-M453), and HtrA4-PD/PDZ (G153-N476) harboring a C-terminal thrombin-cleavable His_6 tag were cloned into a pET-52b vector for bacterial expression. Expression and purification were performed as previously described (7) with slight modifications. Briefly, *E. coli* cultures were grown at 37 °C in LB medium containing 50 μg/mL carbenicillin to an optical density A_{600} of 0.8, after which protein expression was induced with the addition of 0.5 mM IPTG, and grown at 18°C for 24 h. Harvested cell pellets were suspended in lysis buffer (500 mM NaCl, 20 mM Tris pH 8.0, 10% glycerol) and disrupted using a micro-fluidizer. Lysates were clarified by centrifugation (30,000 x g, 30 min) and purified using nickel-nitrilotriacetic acid resin (Qiagen). Resin was washed with 10 column volumes of wash buffer containing 500 mM NaCl, 50 mM Tris pH 8.0, 10% glycerol, 0.1% 3-[(3-cholamidopropyl) dimethylammonio]-1-propanesulfonate (CHAPS), and 50 mM imidazole, followed by elution with the same buffer containing 250 mM imidazole. The eluate was further purified by size exclusion chromatography on a S-200 column (GE Healthcare) in phosphate-buffered saline (PBS) containing 0.1% CHAPS. The elution peak fractions, which corresponded to the trimer forms of the proteins, were collected and their purity assessed by SDS-PAGE.

Anti-HtrA1 antibody generation, humanization, and affinity maturation

We used HtrA1-knockout mice to generate anti-HtrA1 monoclonal antibodies. All knockout ES-cell work was performed using traditional homologous recombination methods and were treated with *cre* recombinase to make full germline knockout lines. We back-crossed the mice to C57Bl6J using speed congenics (>99.8% isogenic based on satellite markers). The mice were immunized with purified muHtrA1-PD. Isolated mice spleen cells were then fused with myeloma cells for hybridoma screening by ELISA protocol. Briefly, 96-well microtiter ELISA plates (Greiner,

Germany) were coated with 2 µg/mL muHtrA1-PD or HtrA1-PD in 0.05 M carbonate buffer (pH 9.6) at 4 °C overnight. After three times of washing with buffer (0.05% Tween 20 in PBS, Sigma), plates were blocked with ELISA diluents (0.5% bovine serum albumin (BSA) and 0.05% Tween 20 in PBS, Sigma) and then incubated with hybridoma cultured supernatants for 1 h incubation at room temperature. The plates were washed three times and incubated with HRP-conjugated goat anti-mouse IgG Fc (Thermo Fisher Scientific, San Jose, CA) for 1 h at room temperature. After washing three times, bound enzyme was detected by tetramethylbenzidine (TMB) substrate (BioFX Laboratories) for 5 min, and the reactions were quenched by stop reagent (BioFX Laboratories), followed by the detection at absorbance 650 nm. As expected from the high sequence homology between human and mouse HtrA1, most of the positive clones were cross-reactive. All ELISA-positive hybridoma clones were tested for inhibitory activity by a fluorescence-based casein cleavage assay (EnzChekTM protease assay kit, Thermo Fisher Scientific, San Jose, CA). Hybridoma supernatants were incubated with HtrA1-PD (final concentration of 20 nM) in 50 mM tris(hydroxymethyl)aminomethane (Tris), 200 mM NaCl, 0.25% CHAPS, pH 8.0 in 96-well plates for 20 min at 37 ^{o}C, then BODIPY FL casein substrate was added and the increase in fluorescence signal (excitation 485 nm, emission 535 nm) was measured at 37 ^{o}C on a SPECTRAmax M5 microplate reader (Molecular Devices), and the linear rate of substrate cleavage over a period of 15 min was determined. The clone 15H6 showed strong inhibitory activity, and its light chain variable region (VL) and the heavy chain variable region (VH) sequences were then obtained by standard RT-PCR approach.

To humanize the 15H6 antibody, the hypervariable regions (HVRs) and all murine framework residues at Vernier zone (36) of the light chain and heavy chain were grafted into the closest human IGKV1 and IGHV1 consensus frameworks, respectively, to generate humanized Fab15H6.v1. In the second step of humanization, key murine framework residues at Vernier zone were identified and retained by the approach as described before (37) to generate Fab15H6.v2, which showed strong inhibitory activity in a fluorescence-quenched peptide substrate assay, which was used

throughout the humanization and affinity maturation process to assess the Fab activities. In the third step of humanization, problematic CDR (complementarity determining region) residues, N94 (CDR-L3) and D55 (CDR-H2), identified by chemical stability analysis (cleavage at N94A-P95 and isomerization of D55), were replaced with A94 (CDR-L3) and E55 (CDR-H2), respectively, to generate Fab15H6.v3.

To improve HtrA1 binding affinity of Fab15H6.v3, to be comparable to Fab15H6.v2, we generated phage-displayed Fab libraries comprising human constant light (CL) and constant heavy 1 (CH1) domains (38), with either light chain (i.e., hypervariable region (HVR)-L1 , HVR-L2, and HVR-L3) or heavy chain HVR residues (i.e., HVR-H1, HVR-H2, and HVR-H3), randomized using NNK degenerate codon, which encode for all 20 amino acids. Libraries were designed to allow one NNK mutation in each of the three light chain or heavy chain hypervariable domains. Phage libraries were panned against decreasing concentration of biotinylated HtrA1-PD in solution for two rounds with 1000-fold molar excess of non-biotinylated HtrA1-PD as competitor in solution to increase the selection stringency for enriching affinity-improved variants.

For deep sequencing phagemid, double stranded DNA was isolated from *E. coli* XL-1 cells carrying phagemid vectors from the initial phage library, and from the 2nd round of selection. Purified DNA was used as template for a limited cycle PCR-based amplification of VL and VH regions using PHUSION® DNA polymerase (New England Biolabs). PCR products were purified by agarose gel extraction and clean-up (Qiagen Gel Extraction Kit). Eluted amplicon DNA was used as the basis for deep sequencing library preparation with standard lllumina library preparation methods, using a TRUSEQ™ DNA Sample Prep kit (lllumina). Adapter-ligated libraries were subjected to a single cycle of PCR and sequenced on the lllumina MISEQ®, using paired-end sequencing with an insert size of 200 bp or 300 bp, as appropriate, to cover the entire length of the amplicon. Deep sequencing data analysis (39) identified 20 highly enriched single mutations and 16 combo mutations. Sequences were synthesized to clone into a mammalian Fab expression construct containing a Flag tag to generate Fab-Flag tag fusion proteins. Plasmids encoding the heavy or light

chain were transfected to 293T cells for 30 mL expression and Fabs were purified with an anti-Flag column.

Fab binding affinity was determined on a BIAcore T200 instrument. Briefly, anti-His tag sensor chip CM5 (GE Healthcare) was generated according to the supplier's instructions to capture HtrA1-PD. For kinetics measurements, 5-fold serial dilutions of each Fab variant (50 nM to 0.8 nM) in HBS-EP buffer (0.01 M 4-(2-hydroxyethyl)piperazine-1-ethanesulfonic acid (HEPES) pH 7.4, 0.15 mM NaCl, 0.003 M EDTA, 0.05% (v/v) surfactant P20) were injected at a flow rate of 50 μL/min at room temperature. The sensorgrams were recorded and subjected to reference and buffer subtraction before analyzing by BIAcore T200 Evaluation Software (version 2.0). Association rates (k_{on}) and dissociation rates (k_{off}) were calculated using a simple one-to-one Langmuir binding model. K_D was calculated as the ratio of k_{off}/k_{on}. The top affinity clone 15H6.v4 had two additional mutations, N31E on CDR-L1 and T28K on CDR-H1, respectively. The flag-tag was deleted to produce the final form of Fab15H6.v4.

Furthermore, in order to evade pre-existing anti-hinge antibodies in human serum against the exposed C-terminal residues of Fab15H6.v4, we truncated the upper hinge region of this Fab (K222-T225) to generate Fab15H6.v4.D221 ending with residue D221 (40). This final Fab was expressed in *E. coli* and purified by affinity chromatography and ion exchange chromatography.

Binding kinetics and specificity assessment by surface plasmon resonance (SPR) of the two anti-HtrA1 Fabs, Fab15H6.v2 and Fab15H6.v4.D221

An anti-His tag sensor chip CM5 (GE Healthcare) was generated according to the supplier's instructions to capture 0.5 μg/mL of HtrA1-PD in HBS-EP buffer (0.01M HEPES pH 7.4 0.15 M NaCl, 0.003 M EDTA, 0.05% (v/v) Surfactant P20) for kinetics measurement. Multiple 5-fold serial dilutions of Fab15H6.v2 and Fab15H6.v4.D221 (100 nM to 0.16 nM) in HBS-EP buffer (0.01 M HEPES pH 7.4, 0.15 M NaCl, 0.003 M EDTA, 0.05% (v/v) Surfactant P20) were prepared and injected at a flow rate of 50 μL/min at room temperature on a BIAcore T200 instrument to obtain

titrated binding response. The sensorgrams were recorded and subjected to reference and buffer subtraction for analysis by BIAcore T200 Evaluation Software (version 2.0). Association rates (k_{on}) and dissociation rates (k_{off}) were calculated using a simple one-to-one Langmuir binding model and the equilibrium dissociation constant (K_D) was calculated as the ratio of k_{off}/k_{on}. For binding specificity assessment, a similar approach as described above was applied. HtrA1-PD/PDZ, HtrA2-PD/PDZ, HtrA3-PD/PDZ, and HtrA4-PD/PDZ at a concentration of 0.5 µg/mL in HBS-EP buffer were first captured by the same anti-His tag sensor chip CM5 on individual flow cell, followed by injection of 100 nM of either Fab15H6.v2 or Fab15H6.v4.D221 or the control Fab (anti-PCSK9 Fab33) (41) in HBS-EP buffer to measure the binding response.

Preparation of Fab15H6.v2 by cleavage of IgG15H6.v2

The sample of IgG15H6.v2 antibody was adjusted to pH 7 with 0.1 M Tris, and Lysyl endoproteinase K (Wako) was added to a concentration of 1 µg enzyme/mg FAb (1:1000), and incubated at 37 ^{o}C for 1 hr. The digest was diluted with 3 volumes of water to reduce the conductivity, and acetic acid was added to a final concentration of 0.2% by volume. The sample was loaded onto a 10 mL SPHP column (GE) equilibrated in 20 mM Na succinate buffer, pH 5.0. The Fab and Fc portions were eluted with a NaCl gradient of 0 to 0.3 M NaCl with 30 column volumes of buffer. The fractions containing the Fab domain (at approximately 0.1 M NaCl) were pooled and filtered through a 0.2 micron filter for storage. Purity of the Fab was assessed by LCMS (Agilent TOF).

Generation of an HtrA1 Specific Activity Based Profiling Probe

The synthesis pathway of an HtrA1 specific activity-based profiling probe is shown schematically below and described in the text.

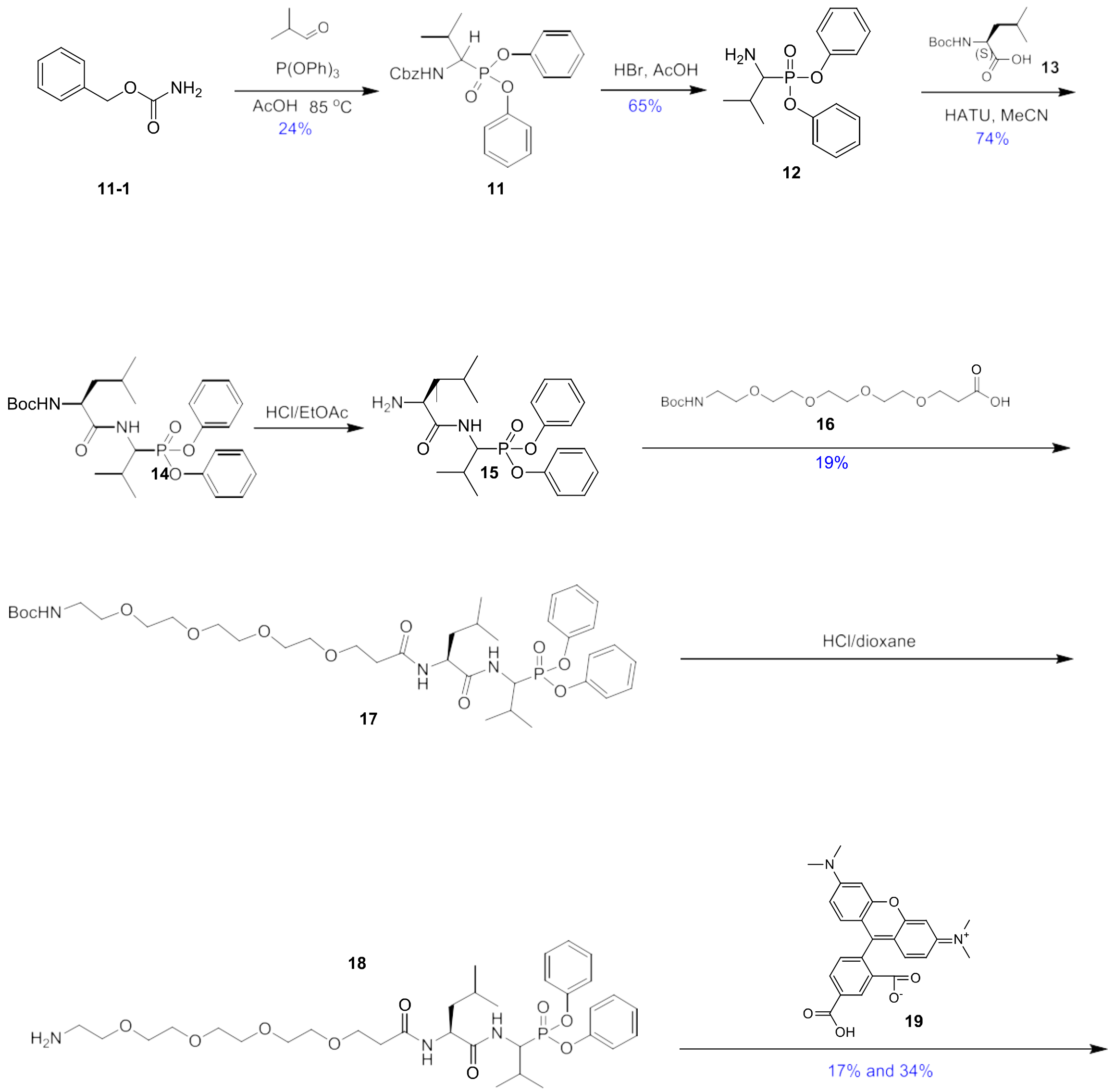

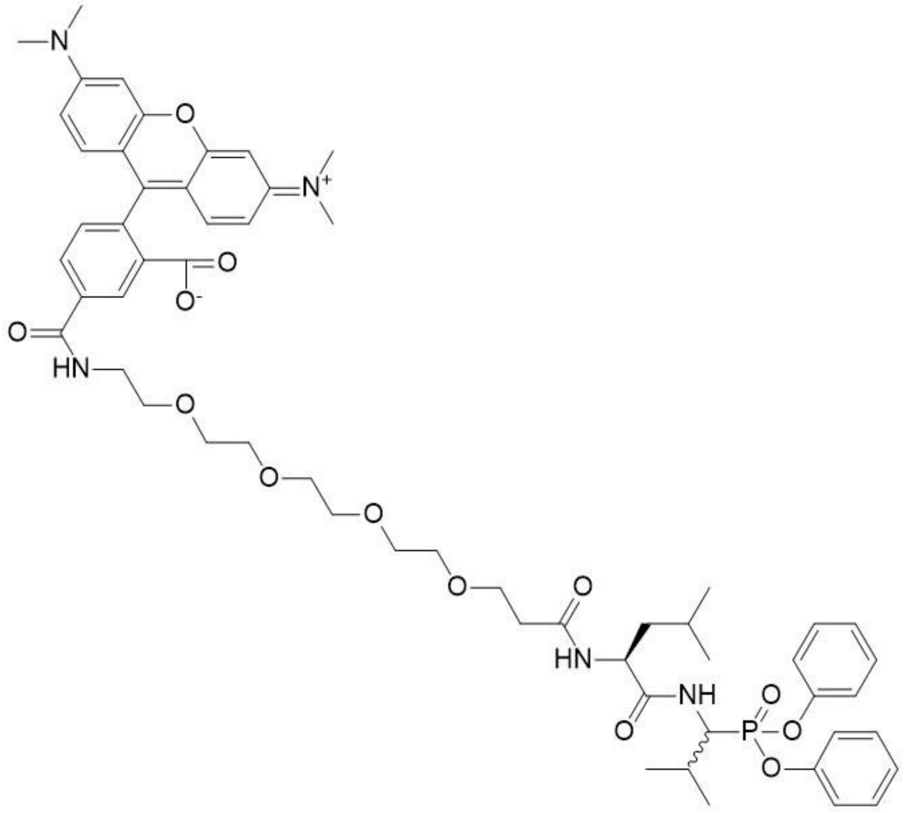

A mixture of 2-methylpropanal (7.16 g, 99.2 mmol, 1.50 eq), **compound 11-1** (10.0 g, 66.2 mmol, 1.00 eq), triphenyl phosphite (20.5 g, 66.2 mmol, 1.00 eq) in AcOH (10.0 mL) was stirred at 85 °C for 4 h. TLC (petroleum ether: ethyl acetate=2/1, R_f = 0.4) showed that the reaction was complete. The reaction mixture was concentrated under reduced pressure to remove solvent. The residue was diluted with MeOH (40 mL). The solution was cooled to −15 °C as a colorless thick oil, which got solidified on standing, and was filtered to give **compound 11** (7.00 g, 15.9 mmol, 24% yield) as a white solid.

A mixture of **Compound 11** (6.00 g, 13.6 mmol, 1.00 eq) in hydrogen bromide (44.2 g, 546 mmol, 40.0 eq) was stirred at 30 °C for 3 h. TLC (Petroleum ether: Ethyl acetate =2/1, R_f = 0.4) showed

that the reaction was complete. The reaction mixture was concentrated under reduced pressure to remove solvent. The residue was washed with methyl tert-butyl ether (MTBE) (10 mL), filtered and concentrated under reduced pressure to give **compound 12** (3.40 g, 8.80 mmol, 65% yield) as a white solid.

A mixture of **compound 12** (1.30 g, 3.37 mmol, 1.00 eq), **compound 13** (934 mg, 4.04 mmol, 1.20 eq), Et_3N (681 mg, 6.73 mmol, 2.00 eq), hexafluorophosphate azabenzotriazole tetramethyl uronium (HATU) (1.54 g, 4.04 mmol, 1.20 eq) in MeCN (10.00 mL) was degassed and purged with N_2 3 times, and the mixture was then stirred at 30 °C for 18 h under N_2 atmosphere. LCMS showed that the reaction was complete. The reaction mixture was quenched with H_2O (5 mL) and diluted with EtOAc (5 mL). The resulting mixture was extracted with EtOAc (5 mL *3). The combined organic layers were washed with brine (3 mL), dried over Na_2SO_4, filtered, and concentrated under reduced pressure to give a residue. The residue was purified by column chromatography (SiO_2, Petroleum ether/Ethyl acetate=10/1 to 5:1). **Compound 14** (1.30 g, 2.51 mmol, 74% yield) was obtained as a white solid.

A mixture of **compound 14** (1.00 g, 1.93 mmol, 1.00 eq) in HCl/dioxane(20 mL) was stirred at 30 °C for 1 h. TLC (petroleum ether: ethyl acetate=2/1, R_f = 0.1) showed that the reaction was complete. The reaction mixture was concentrated under reduced pressure to remove dioxane. The crude product **compound 15** (1.00 g, crude) was used into the next step without further purification.

A mixture of **compound 15** (1.00 g, 2.04 mmol, 1.00 eq), Et_3N (826 mg, 8.16 mmol, 4.00 eq), **compound 16** (745 mg, 2.04 mmol, 1.00 eq), and HATU (929 mg, 2.44 mmol, 1.20 eq) in MeCN

(10 mL) was degassed and purged with N_2 3 times, and the mixture was then stirred at 30 °C for 2 h under N_2 atmosphere. LC-MS showed that the reaction was complete. The reaction mixture was concentrated under reduced pressure to remove the solvent. The residue was purified by prep-HPLC (TFA condition). **Compound 17 (SS or SR)** (450 mg, 587 µmol, 28.8% yield) and **compound 17A (SS or SR)** (300 mg, 392 µmol, 19.2% yield) were obtained in the form of oil.

A mixture of **compound 17 or 17A** (50 mg, 65 µmol, 1.00 eq) in HCl/dioxane (500 µL was stirred at 15 °C for 2 h. LCMS showed that the reaction was complete. The reaction mixture was concentrated under reduced pressure. The crude product **compound 18 or 18A** (50 mg, crude) was used into the next step without further purification.

A mixture of **compound 18 or 18A** (50 mg, 75.1 µmol, 1.00 eq), **compound 19** (35.6 mg, 82.6 µmol, 1.10 eq), HBTU (34.2 mg, 90.1 µmol, 1.20 eq), and DIPEA (29.1 mg, 225.3 µmol, 3.00 eq) in MeCN (1.60 mL) was degassed and purged with N_2 3 times, and the mixture was then stirred at 30 °C for 18 h under N_2 atmosphere. LCMS showed that the reaction was complete (**Fig S2b**). The solution was purified by prep-HPLC (neutral condition). Final product was assessed by NMR (**Fig S2c**).

Supplemental Figures:

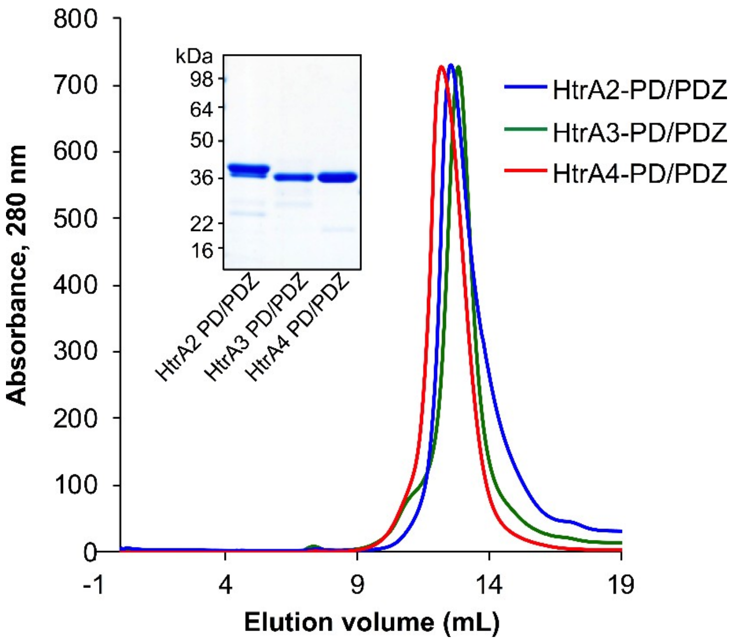

Fig. S1a: Purification of PD/PDZ constructs of HtrA2, 3, and 4.

The *E. coli* expressed proteins were purified from lysates using nickel-nitrilotriacetic acid resin chromatography. The column eluates were further purified by size exclusion chromatography on a S-200 column shown here. The elution peaks of the proteins are consistent with PD/PDZ trimers, and SDS-PAGE analysis (non-reducing conditions) confirmed the purity of the proteins.

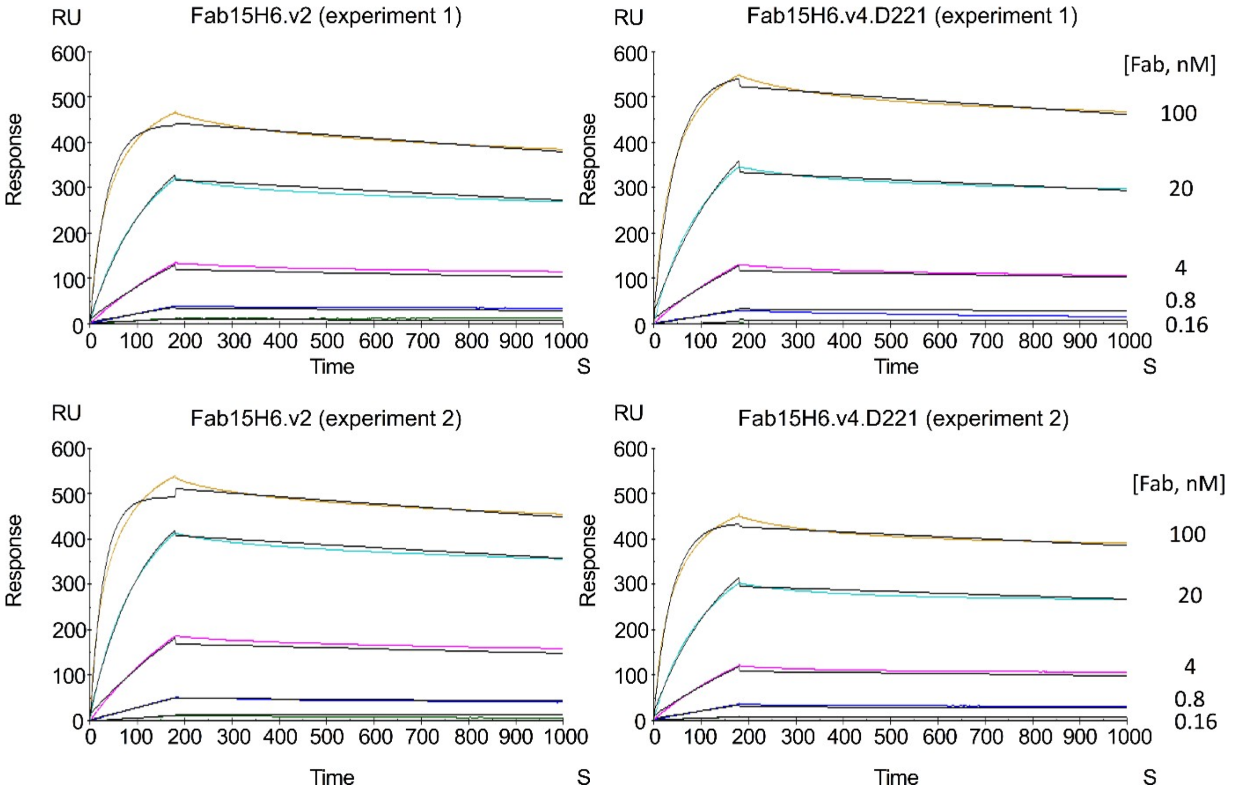

Fig. S1b: Binding kinetics of Fab15H6.v2 and Fab15H6v4.D221 by SPR

Binding kinetics of Fab15H6.v2 and Fab15H6.v4.D221 was measured by surface plasmon resonance. The His-tagged trimer of HtrA1-PD was captured by anti-His tag sensor chip CM5 on a BIAcore T200 instrument, followed by injection of serially diluted Fab15H6.v2 or Fab15H6.v4.D221 (100-20-4-0.8-0.16 nM). Shown are the sensorgrams of two individual experiments for each Fab. The K_D values were calculated from the k_{on} and k_{off} values ($K_D = k_{off}/k_{on}$), which were determined by using a simple one-to-one Langmuir binding model. The two K_D values for Fab15H6.v2 were 0.671 nM and 0.446 nM, respectively (average = 0.559 nM) and those for Fab15H6.v4.D221 were 0.699 nM and 0.477 nM (average = 0.588 nM).

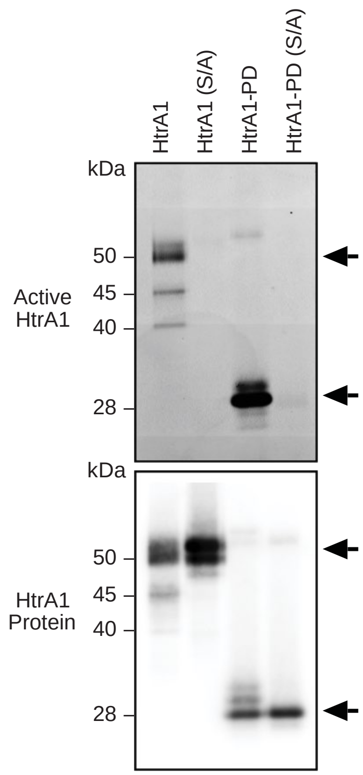

Fig. S2a: The HtrA1 activity-based probe was reactive against HtrA1 and HtrA1-PD, but did not bind the respective Ser to Ala catalytically inactive mutants, demonstrating its specificity to the catalytically active version of this enzyme. Arrows indicate intact HtrA1 or HtrA1-PD on fluorescent gel images (*top panel*) or immunoblots (*bottom panel*), respectively.

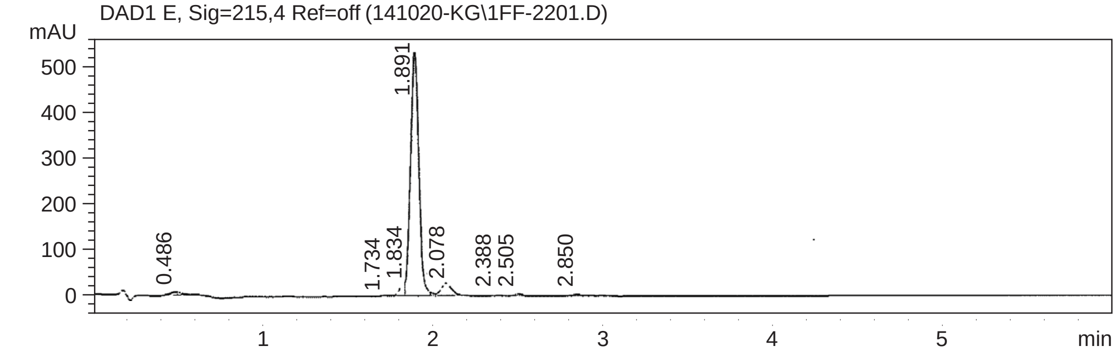

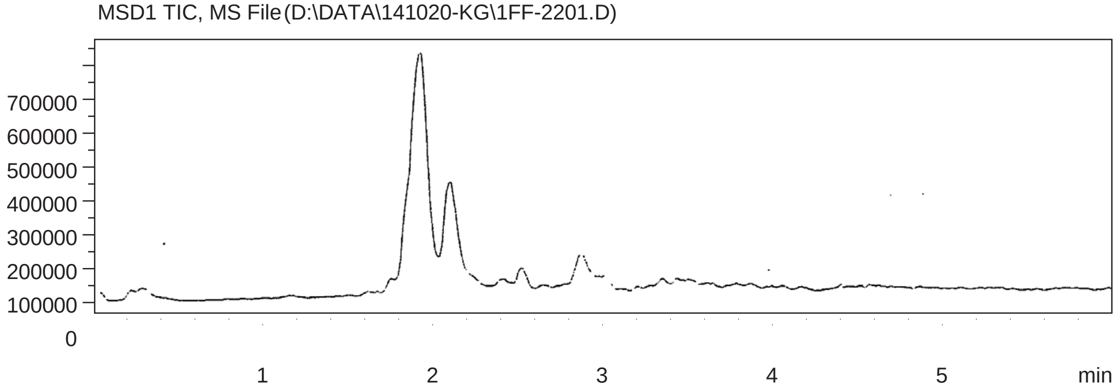

Integration Result

Peak #	RT (min)	Area	Height	Height %	Width (min)	Area %
1	0.486	27.864	5.683	0.924	0.082	1.348
2	1.734	6.410	2.098	0.341	0.051	0.310
3	1.834	63.300	28.935	4.703	0.036	3.062
4	1.891	1802.318	542.040	88.106	0.055	87.198
5	2.078	127.180	27.684	4.500	0.077	6.153
6	2.388	4.602	1.454	0.236	0.053	0.223
7	2.505	12.910	4.324	0.703	0.050	0.625
8	2.850	22.352	2.996	0.487	0.124	1.081

Fig. S2b: LC/MS Analysis of HtrA1 ABP

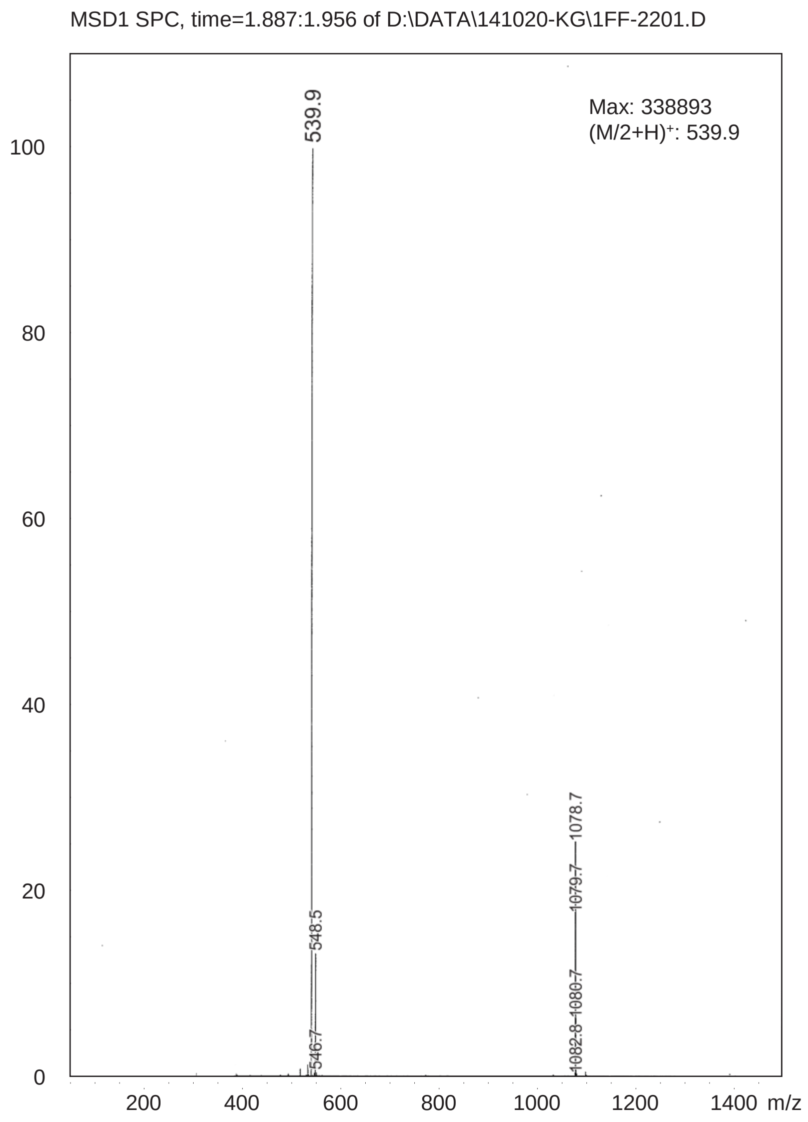

Fig.S2c: MS spectrum of HtrA1 ABP

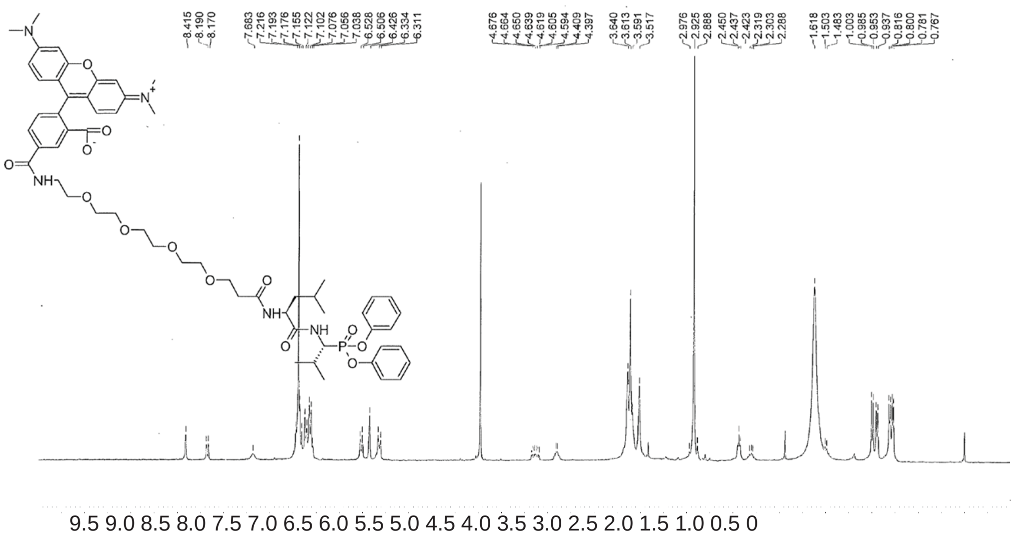

^{1}H NMR: ET2896-56-P1 400 MHz CDCl3
δ 7.41 (d, J = 2.6 Hz, 1H), 7.44 - 7.55 (m, 3H), 7.63 (d, J = 8.8 Hz, 2H), 7.94 (s, 1H), 8.26 (s, 1H), 8.64 (d, J = 5.3 Hz, 1H), 8.95 (d, J = 2.2 Hz, 1H), 9.34 (s, 1H)
d ppm 8.36 (s, 1 H), 8.15 (d, J =8.82 Hz, 1H), 7.48 (br. s., 1H), 7.19 (s, 17H), 7.09 - 7.15 (m, 3H), 7.04 (d, J =7.50 Hz, 4H), 6.90 - 7.00 (m, 1H), 6.39 - 6.54 (m, 4H), 6.29 (t, J =9.26 Hz, 2H), 4.60 - 4.71 (m, 1H), 4.53 (br. s., 1H), 3.53 - 3.70 (m, 16H), 3.39 - 3.51 (m, 6H), 2.89 - 2.99 (m, 12H), 2.28 - 2.42 (m, 3H), 1.52 (br. s., 35H), 0.98 - 1.05 (m, 7H), 0.83 (dd, J =8.60, 6.39 Hz, 7H)

Fig.S2d: ^{1}H NMR spectrum of HtrA1 ABP

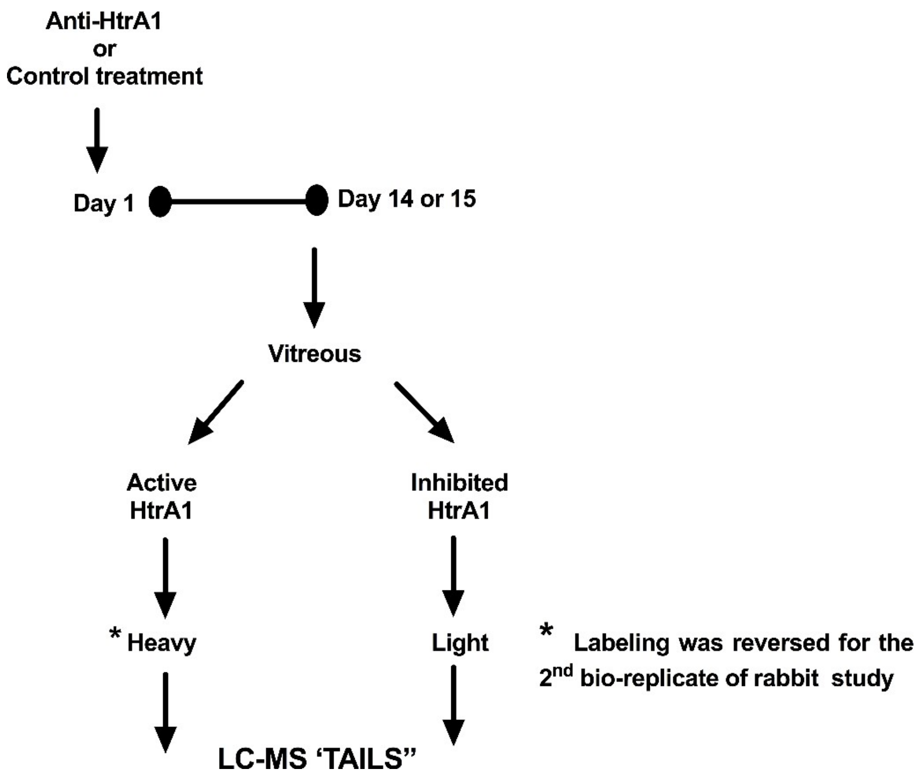

Fig. S3. Terminal Amine Isotopic Labeling of Substrates (TAILS) was performed using vitreous humor harvested from rabbits post treatment with anti-HtrA1 IgG15H6.v2 (0.2 mg) for 14 days or monkeys post treatment with Fab15H6.v4.D221 (0.2 mg and 6 mg) for 15 days (HtrA1- inhibited) vs. control treatment (HtrA1-active).

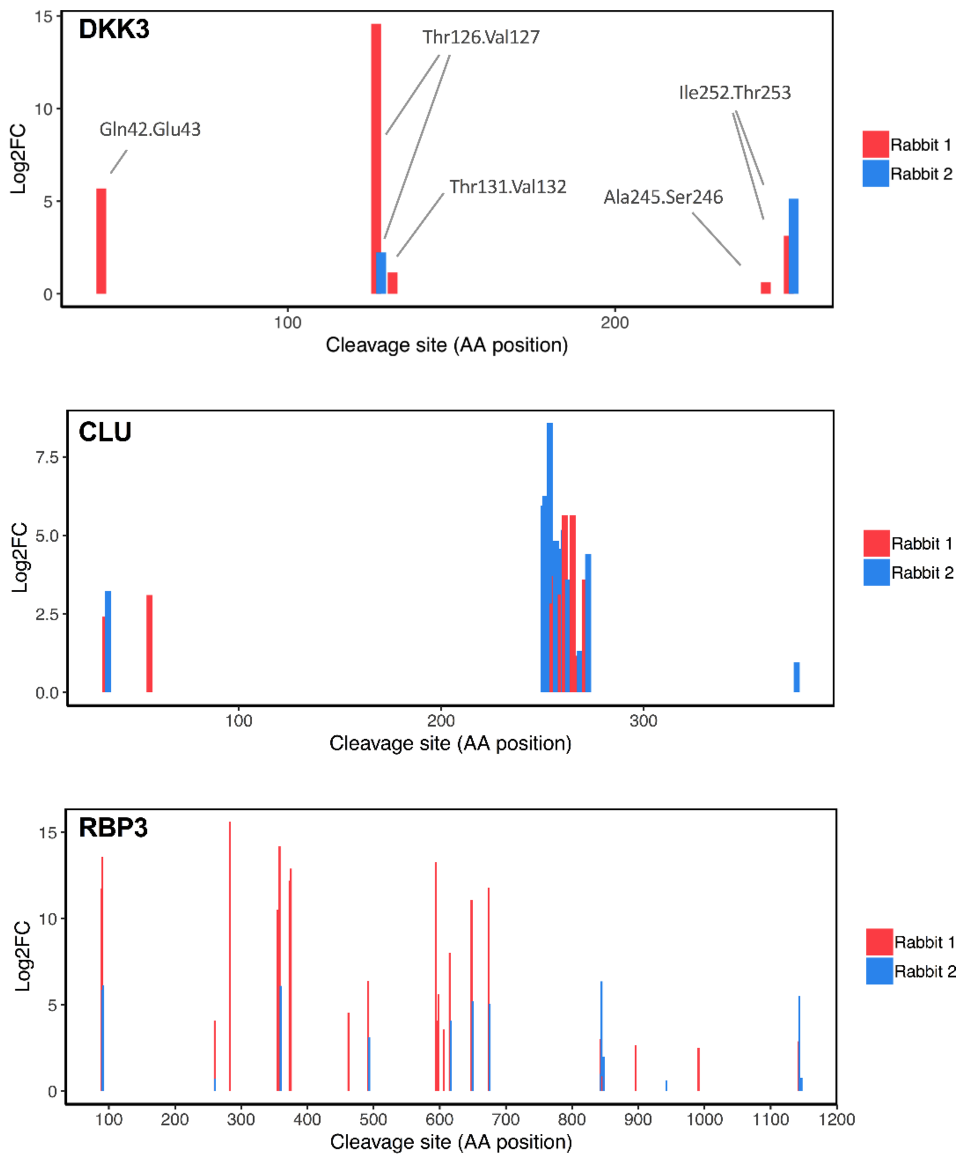

Fig. S4: Amino acid position of HtrA1 cleavage sites identified by TAILS for DKK3, CLU, and RBP3.

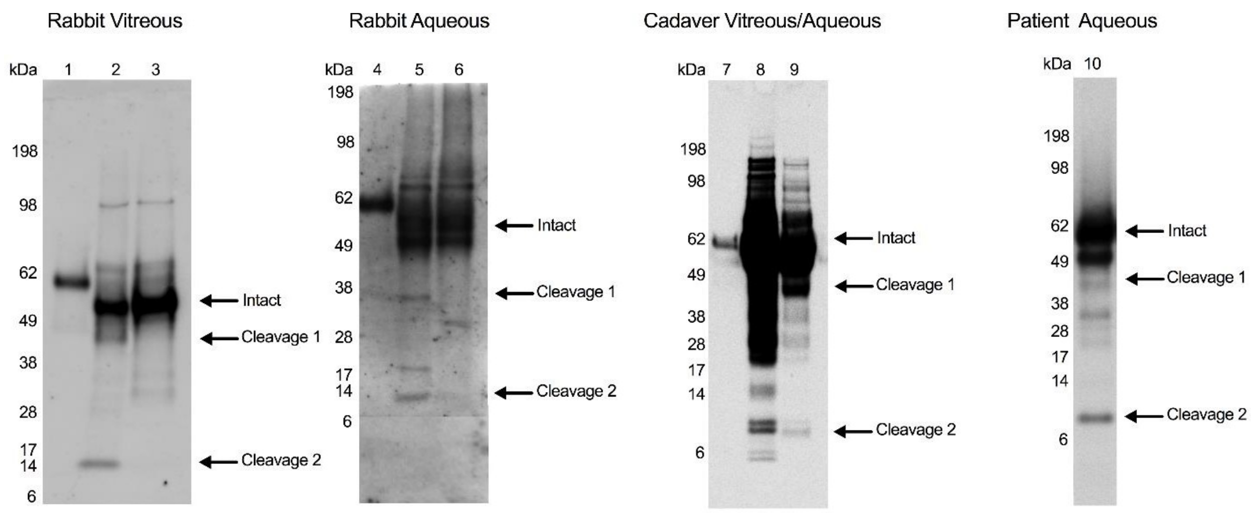

Fig. S5: Validation of *in vivo* cleavage of DKK3 in rabbits, human cadaver, and AMD patient. Western blot analysis of rabbits treated with anti-gD control antibody (Lanes 2 and 5) or anti-HtrA1 IgG15H6.v2 (Lanes 3 and 6) identified HtrA1 specific cleavage products of DKK3 in vitreous and aqueous humor. Similar bands were observed in cadaver vitreous (Lane 8) and aqueous humor (Lane 9) and in patient aqueous humor (Lane 10, Subject ID:10003 at baseline). Recombinant DKK3 (5 nM) was used as control in Lanes 1, 4, and 7. Arrows indicate intact and cleavage 1 and 2 as shown in Fig. 5A.

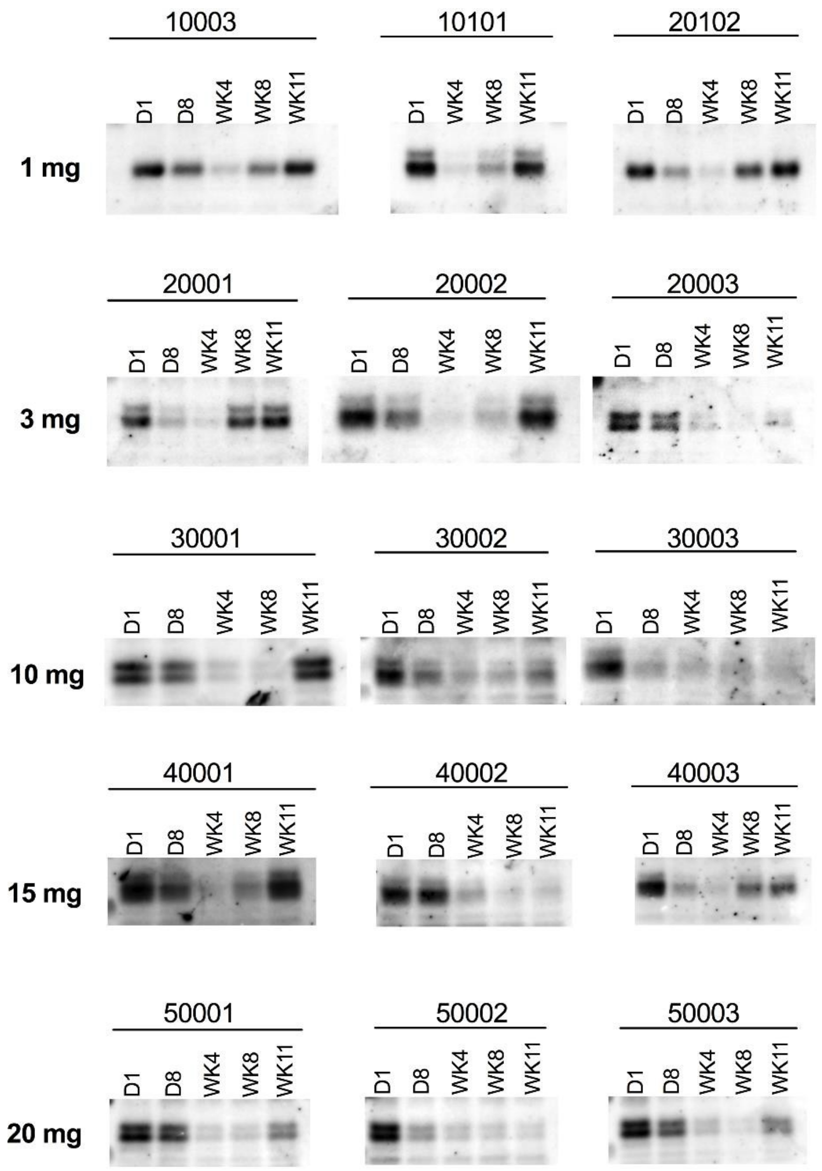

Fig. S6: Western blot analysis of SAD patient aqueous humor revealed dose dependent inhibition of DKK3 cleavage by anti-HtrA1 Fab15H6.v4.D221. Aqueous humor was monitored at baseline and post-administration with anti-HtrA1 Fab15H6.v4.D221. Quantification of DKK3 cleaved bands was performed by gel densitometric analysis.

Supplemental Tables:

Table S1: List of all Neo N-terminal peptides that were quantified in 2 rabbit and 1 cynomolgus TAILS experiments. The intensity of the peptides that were not identified in one condition was imputed to the lowest signal intensity reported in that condition.

Identifier.rabbit	Reference	Description	Gene	log2Active2 Inhibited. TAILS1	log2Active 2Inhibited. TAILS2	Gene. Group	Imputed	Overlay .cyno	TAILS. Cyno.lo g2Activ	Log2F C. Impute	Size
GRDFYKILGVPR	G1SGQ0_RABIT	UP	DNAJB11	2.75556152	1.149283327	Other	FALSE	No	NA	No	6
TPNVEFITGGPDDR	CLUS_RABIT	CLU	CLU	1.170995214	1.327782025	CLU	FALSE	No	NA	No	6
THLHVIEER	G1SYG6_RABIT	UP	APLP2	3.072803006	1.765891318	Other	FALSE	No	NA	No	6
SGSAAEAFAHTMQDLQR	G1T5S0_RABIT	UP	RBP3	0.856082976	2.011182532	RBP3	FALSE	No	NA	No	6
VFSETVITSVGDEEGKR	G1SMM4_RABIT	UP	DKK3	14.58007763	2.230168679	DKK3	TRUE	No	NA	Yes	6
SYFQGPEDSPVR	G1T5S0_RABIT	UP	RBP3	6.389722962	3.123277816	RBP3	FALSE	No	NA	No	6
STQGSKYIDR	CLUS_RABIT	CLU	CLU	2.407389384	3.231155007	CLU	FALSE	No	NA	No	6
HSPAYQTPNVEFITGGPDDR	CLUS_RABIT	CLU	CLU	5.635659054	3.599931602	CLU	FALSE	No	NA	No	6
NAGLQSVSEDPR	G1T5S0_RABIT	UP	RBP3	12.19149489	3.952298258	RBP3	TRUE	No	NA	Yes	6
VLAEEALDR	G1T5S0_RABIT	UP	RBP3	8.025761633	4.075296002	RBP3	FALSE	No	NA	No	6
EFITGGPDDR	CLUS_RABIT	CLU	CLU	3.589495911	4.398971387	CLU	FALSE	No	NA	No	6
MDVHLHSPAYQTPNVEFITGGPDDR	CLUS_RABIT	CLU	CLU	3.70833721	4.57720032	CLU	FALSE	Yes	2.107	No	9
AMDVHLHSPAYQTPNVEFITGGPDDR	CLUS_RABIT	CLU	CLU	2.825189221	4.832264323	CLU	FALSE	No	NA	No	6
AVDLESLASQLTADLQEVSGDHR	G1T5S0_RABIT	UP	RBP3	11.79598951	5.047886694	RBP3	TRUE	Yes	3.792	Yes	9
TWELEPDGALDR	G1SMM4_RABIT	UP	DKK3	3.129406657	5.132562074	DKK3	FALSE	Yes	0.604	No	9
HLHSPAYQTPNVEFITGGPDDR	CLUS_RABIT	CLU	CLU	3.109524942	5.176047504	CLU	FALSE	No	NA	No	6
HYARPEVVGQASALLR	G1T5S0_RABIT	UP	RBP3	11.07518173	5.216946927	RBP3	TRUE	No	NA	Yes	6
SSLTAGAAEEFTYIMKR	G1T5S0_RABIT	UP	RBP3	2.892958147	5.502502056	RBP3	FALSE	No	NA	No	6
MNEVQDHLEEEER	G1SVL6_RABIT	CLU	CLUL1	8.388459312	5.503657951	Other	TRUE	No	NA	Yes	6
ISYEPSTLEVPR	G1T5S0_RABIT	UP	RBP3	11.75864472	5.867408633	RBP3	TRUE	No	NA	Yes	6
DFSTVVSEEDLVSKLNAGLQSVSEDPR	G1T5S0_RABIT	UP	RBP3	14.2124082	6.105259123	RBP3	TRUE	No	NA	Yes	6
SYEPSTLEVPR	G1T5S0_RABIT	UP	RBP3	13.57312523	6.121174825	RBP3	TRUE	No	NA	Yes	6
SHTSGSAAEAFAHTMQDLQR	G1T5S0_RABIT	UP	RBP3	3.011038445	6.366202995	RBP3	TRUE	No	NA	Yes	6
YFDAVQAIETHLIR	MA1A1_RABIT	MOM1A	MAN1A1	5.505125759	6.455956624	Other	TRUE	No	NA	Yes	6
HAFDSTPNLKGIFLR	G1SHT7_RABIT	UP	PODN	13.92822827	7.618948571	Other	TRUE	No	NA	Yes	6
EDPAPPQWDWR	A5HC46_RABIT	Cathepsin F	A5HC46_ProtAcs	16.13772212	8.263516466	Other	TRUE	No	NA	Yes	6

TAILS, Terminal amine isotopic labeling of substrates; MOM1A, mannosyl-oligosaccharide 1,2-alpha-mannosidase IA; UP, uncharacterized protein; CLU, clusterin; RBP3, retinol binding protein 3; DKK3, Dickkopf related protein 3

Table S2: Percent Change from Baseline for Aqueous Humour DKK3 and Calculated Mean and Mean Change from Baseline by Visit, Safety-Evaluable Patients

Dickkopf Related Protein 3 Cleavage Product Immunoblot Band Densitometry in Relative Luminescence Units (RLU)

		**Fab15H6.v4.D221 1 mg (N=3)**			**Fab15H6.v4.D221 3 mg (N=3)**			**Fab15H6.v4.D221 10 mg (N=3)**			**Fab15H6.v4.D221 15 mg (N=3)**			**Fab15H6.v4.D221 20 mg (N=3)**	
**Visit**	**Subject ID**	**Value at Visit**	**% Change from Baseline**	**Subject ID**	**Value at Visit**	**% Change from Baseline**	**Subject ID**	**Value at Visit**	**% Change from Baseline**	**Subject ID**	**Value at Visit**	**% Change from Baseline**	**Subject ID**	**Value at Visit**	**% Change from Baseline**
**Baseline**															
	20102	5025704		20001	4248474		30002	15914260		40001	21444331		50001	16537430	
	10101	11740430		20002	6973279		30001	22769117		40002	14507433		50003	25858699	
	10003	8308019		20003	1363432		30003	6167390		40003	17878585		50002	19536012	
	Mean (SD)	8358051.0 (3357642.6)		Mean (SD)	4195061.7 (2805304.9)		Mean (SD)	14950255.7 (8342740.1)		Mean (SD)	17943449.7 (3468903.9)		Mean (SD)	20644047.0 (4758394.7)	
**Day 8**															
				20001	1141990	-73.1	30002	9338216	-41.3	40001	12381935	-42.3	50001	12061702	-27.1
	20102	1750166	-65.2	20002	3591228	-48.5	30001	15374201	-32.5	40002	13028488	-10.2	50003	16677717	-35.5
	10003	5087301	-38.8	20003	1053990	-22.7	30003	1149652	-81.4	40003	6924893	-61.3	50002	5872864	-69.9
	Mean (SD)	3418733.5 (2359710.8)	**-52.0 (18.7)**	Mean (SD)	1929069.3 (1440143.9)	**-48.1 (25.2)**	Mean (SD)	8620689.7 (7139368.4)	**-51.7 (26.0)**	Mean (SD)	10778438.7 (3352889.6)	**-37.9 (25.8)**	Mean (SD)	11537427.7 (5421472.1)	**-44.2 (22.7)**
**Week 4**															
	20102	431293	-91.4	20001	290324	-93.2	30002	4644259	-70.8	40001	3865103	-82	50001	2080078	-87.4
	10101	795741	-93.2	20002	162822	-97.7	30001	3592048	-84.2	40002	4102656	-71.7	50003	5306295	-79.5
	10003	1005975	-87.9	20003	175565	-87.1	30003	693254	-88.8	40003	4656930	-74	50002	2934637	-85
	Mean (SD)	744336.3 (290769.1)	**-90.8 (2.7)**	Mean (SD)	209570.3 (70224.4)	**-92.7 (5.3)**	Mean (SD)	2976520.3 (2046158.8)	**-81.3 (9.4)**	Mean (SD)	4208229.7 (406333.4)	**-75.9 (5.4)**	Mean (SD)	3440336.7 (1671501.8)	**-84.0 (4.1)**
**Week 8**															
	20102	3972009	-21	20001	4415427	3.9	30002	3828202	-75.9	40001	5511630	-74.3	50001	2204891	-86.7
	10101	4112922	-65	20002	819428	-88.2	30001	3247646	-85.7	40002	1728546	-88.1	50003	2884875	-88.8
	10003	3937276	-52.6	20003	42200	-96.9	30003	1167849	-81.1	40003	10272738	-42.5	50002	2409813	-87.7
	Mean (SD)	4007402.3 (93018.3)	**-46.2 (22.7)**	Mean (SD)	1759018.3 (2333109.7)	**-60.4 (55.9)**	Mean (SD)	2747899.0 (1398813.7)	**-80.9 (4.9)**	Mean (SD)	5837638.0 (4281415.1)	**-68.3 (23.4)**	Mean (SD)	2499859.7 (348820.7)	**-87.7 (1.1)**
**Week 11**															
	20102	5927904	18	20001	5717802	34.6	30002	8098573	-49.1	40001	18574822	-13.4	50001	6988256	-57.7
	10101	9907091	-15.6	20002	7176612	2.9	30001	24226804	6.4	40002	2370719	-83.7	50003	11523706	-55.4
	10003	8040014	-3.2	20003	318981	-76.6	30003	1177619	-80.9	40003	10705171	-40.1	50002	1844484	-90.6
	Mean (SD)	7958336.3 (1990850.5)	**-12.3 (7.9)**	Mean (SD)	4404465.0 (3612536.0)	**-13.0 (57.3)**	Mean (SD)	11167665.3 (11827118.3)	**-41.2 (44.2)**	Mean (SD)	10550237.3 (8103162.5)	**-45.72 (35.5)**	Mean (SD)	6785482.0 (4842795.9)	**-68.0 (19.6)**

Other supplementary materials for this manuscript include the following:

Dataset S1: Neo N-terminal peptides that were elevated in active HtrA1 versus inactive counterpart in both rabbit TAILS analyses. For Neo N-terminal peptides that were not identified in one sample, the intensity was imputed to the lowest signal intensity reported in that sample.
